# 3-CMC: Acute Effects in Male and Female Mice, Human Intoxication Case Series (Italy, 2014–2025), and Prediction of ADMET Properties

**DOI:** 10.3390/ijms262311600

**Published:** 2025-11-29

**Authors:** Marta Bassi, Elisa Roda, Giorgia Corli, Sabrine Bilel, Fabrizio De Luca, Tatiana Bernardi, Adolfo Gregori, Fabiana Di Rosa, Davide Lonati, Carlo Alessandro Locatelli, Matteo Marti

**Affiliations:** 1 Department of Translational Medicine, Section of Legal Medicine, LTTA Center and University Center of Gender Medicine, University of Ferrara, 44121 Ferrara, Italy; marta.bassi@unife.it (M.B.); giorgia.corli@unife.it (G.C.); sabrine.bilel@unife.it (S.B.); 2 Laboratory of Clinical & Experimental Toxicology, Toxicology Unit, Pavia Poison Centre and National Toxicology Information Centre, Istituti Clinici Scientifici Maugeri IRCCS, 27100 Pavia, Italy; elisa.roda@icsmaugeri.it (E.R.); davide.lonati@icsmaugeri.it (D.L.); carlo.locatelli@icsmaugeri.it (C.A.L.); 3 Department of Biology and Biotechnology “Spallanzani”, University of Pavia, 27100 Pavia, Italy; fabrizio.deluca01@universitadipavia.it; 4 Department of Environmental Sciences and Prevention, University of Ferrara, 44121 Ferrara, Italy; tatiana.bernardi@unife.it; 5 Department of Scientific Investigation (RIS), Carabinieri, 00191 Rome, Italy; 6 Department of Scientific Investigation (RIS), Carabinieri, 98122 Messina, Italy; 7 Collaborative Center for the Italian National Early Warning System (NEWS-D), Department of Anti-Drug Policies and Other Addictions, Presidency of the Council of Ministers, 00186 Rome, Italy

**Keywords:** 3-CMC, chloro-methcathinones, synthetic cathinones, human intoxications, pharmacokinetic, Novel Psychoactive Substances

## Abstract

3-chloromethcathinone (3-CMC) is a synthetic cathinone that gained relevance, having been involved in a large number of seizures and poisoning reports. Despite this, literature currently lacks information on its pharmaco-toxicological effects. This study aims to investigate the acute sensorimotor and physiological effects of 3-CMC (0.1–30 mg/kg; i.p.) in male and female CD-1 mice and its effects (1 and 10 mg/kg) on Prepulse Inhibition (PPI). Furthermore, we describe a series of 3-CMC (or CMC)-related human intoxications (Italy, 2014–2025) registered by the PCC–National Toxicology Information Centre. Finally, we predicted the ADMET properties of 3-CMC compared to 2-CMC, 4-CMC, 2-MMC, and two 3-CMC metabolites. 3-CMC induced in mice locomotor stimulation in mice, relevant tachypnoea and hypothermia, sensorimotor, and PPI alterations were observed only at high doses, with minor sex differences. All intoxications were non-fatal and involved male patients showing psychomotor agitation, psychosis, aggressiveness, CNS depression, but also cardiac arrhythmias, thoracic pain, and tachypnea. N-dealkylation, N-hydroxylation, and phenyl hydroxylation were the main predicted reactions. Drug–drug interaction potential and cardiotoxicity were suggested for all compounds. This interdisciplinary study elucidates 3-CMC effects and its associated risks, opening new objectives for future studies on CMC compounds to provide critical information to clinicians and the toxicological field.

## 1. Introduction

The illicit drug market represents a dynamic context where Novel Psychoactive Substances (NPS) are becoming more and more accessible. Indeed, in little over twenty years, almost 1000 NPS were monitored by the European Monitoring Centre for Drugs and Drug Addiction (EMCDDA). Among the different classes of NPS, that of Synthetic Cathinones (SCs) is one of the most frequently reported or seized in Europe [1,2]. SCs are available in the form of pills, powder, or capsules, and are usually present in products sold not for human consumption and labeled as “bath salts” or “plant food”. They are mainly trafficked from India and are sold alongside or passed off as other drugs and often used as adulterants [3]. SCs are designed from the chemical structure of cathinone, a psychoactive compound present in the leaves of Catha edulis, and similarly to cocaine or amphetamine, they induce stimulant effects via the interaction at monoamine transporters responsible for the uptake of dopamine (DA), noradrenaline (NE), and serotonin (5-HT) [4]. They cause euphoria, increased energy, fatigue reduction, and appetite suppression, and are usually consumed in association with other substances and in social situations [5].

**Figure 1:**
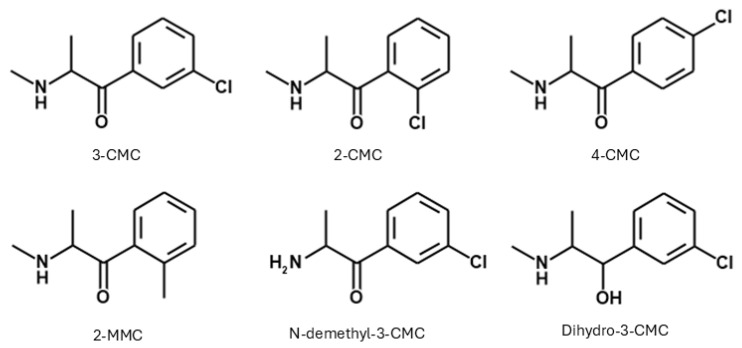
Chemical structure of the synthetic cathinones 3-chloromethcathinone (3-CMC), 2-chloromethcathinone (2-CMC), 4-chloromethcathinone (4-CMC), 2-methylmethcathinone (2-MMC), N-demethyl-3-CMC, and dihydro-3-CMC.

The high number of seizures led to SCs, more specifically, chloro-methcathinones (CMCs) and methyl-methcathinones (MMCs), gaining increasing relevance. In particular, 3-chloro-methcathinone (3-CMC; Figure 1), 4-chloro-methcathinone (4-CMC; Figure 1), and 2-methyl-methcathinone (2-MMC; Figure 1) were reported among the top five SCs seized in 2022. Additionally, seizures of small quantities of 2-chloro-methcathinone (2-CMC; Figure 1) and precursors for its production were described [6,7].

### 3-CMC and Its Effect on Humans

Since 2014, the year of its first detection, 90% of all 3-CMC seizures occurred between 2020 and 2021, coinciding with the ban of 4-CMC. It was the most seized NPS in Europe during 2021 and 2022, representing 34% and 63.41% of total material, respectively [1,2]. 3-CMC is normally sold in the racemic form, mostly in powder or crystals but also as tablets, capsules, liquids, herbal material, and blotters [8]. Similarly to other SCs [3], it is consumed by oral, nasal, or parenteral route [1] and, as reported by users, the doses vary from 20 to 250 mg based on the route of administration [9].

It is known that 3-CMC is a monoamine releaser agent [10], but its effects still need to be clarified, as the current knowledge derives from users’ personal experiences. The described side effects align with the class of SCs [3] and include cardiovascular effects, irritability, compulsive redosing, involuntary and rhythmic movement of the tongue, bruxism, confusion, and memory loss [9].

Table 1 lists the intoxication case reports (both non-fatal, part A, and fatal, part B) involving 3-CMC (or CMC) currently available in the literature. In addition, a total of 161 cases involving 3-CMC were reported between 2015 and August 2023 [8]. Eight fatal intoxications involving 3-CMC were described in Sweden between 2018 and 2022; in seven cases, the drug was considered the cause of death, while in the other case, the drug was listed as contributing to death. In 38 cases of drug-impaired driving, 3-CMC was confirmed in blood and/or urine. Several physiological and adverse effects reported after 3-CMC poisoning included vomiting, headache, large pupils, hyperventilation, agitation, motor restlessness, sweating, increased pulse, high blood pressure, chest pain, and seizures [8]. Exposure to 3-CMC was confirmed in an acute non-fatal poisoning in Spain in one subject, related to ChemSex practices, and additional drugs were detected during testing of biological samples, including 3-MMC, GHB/GLB, cocaine, sildenafil, and methamphetamines. No further details were provided. Moreover, several cases characterized by serious adverse effects were reported in France and Sweden, even though the analytical confirmation was completely lacking. These comprise one case of drug dependence reported by France and 47 acute poisonings with suspected 3-CMC exposure referred to the Swedish Poisons Information Centre between 2015 and 2021 [6].

Currently, the main studies on 3-CMC focus on its metabolism, its analytical determination from biological samples, and its discrimination from its isomers, as the identification of the specific structural isomer in biological fluids can be difficult [11]. Moreover, there is limited information on its acute toxicity, especially when taken alone. Thus, after the seizure of 3-CMC by authorities on Italian territory, the present study aims at investigating its acute behavioral, sensorimotor, and physiological (breath rate and body temperature) effects on female and male CD-1 mice. To provide useful information from a translational perspective, we also present clinical data about intoxications involving 3-CMC that occurred in Italian territory and were collected by the Pavia Poison Control Centre (PCC)–National Toxicology Information Centre (Istituti Clinici Scientifici Maugeri, IRCCS Pavia, Italy) between 2014 and 2025. Moreover, we predicted and compared the ADMET profile of 3-CMC, 2-CMC, 4-CMC, and 2-MMC by using the ADMET Predictor^®^, to highlight differences in the pharmacokinetic properties of analogues and structural isomers, including compounds not under international control. Indeed, at present, among the tested compounds, only 4-CMC and 3-CMC are scheduled in the 1971 Convention on Psychotropic Substances. Additionally, we tested two 3-CMC metabolites (N-demethyl-3-CMC and dihydro-3-CMC; Figure 1) to evaluate their toxicity potential.

**Table 1:** Literature data on non-fatal (Part A) and fatal (Part B) human poisonings: clinical presentation of patients with analytically confirmed CMC use in blood/urine samples.

*Part A*								
*Case n./Sex, Age*	*Hospital* *Admission/* *Outcome*	*Self-Reported Substance(s)*	*Reported Route of Intake*	*Signs/Symptoms* *on Medical Examination*	*Patient Management*	*Blood/Urine/Substance Analytical Results*	*Anamnestic Data,* *Annotations,* *Hospital Stay/Outcomes*	*References*
#**1** 16, F	*Emergency/* *non-fatal*	Legal high preparation, named HEX-EN	Intranasal	CNS alterations, i.e., psychomotor inhibition, psychosis, bradycardia, hypotension, bradypnea, minimal Paediatric GCS point: 8. Muscle stiffness	Symptomatic treatment	Legal high preparation: 3-CMC, HEX-EN (n-ethylhexedrone), MAB-CHMINACA, and caffeine	Admitted to Dept of Paediatrics and Clinical assessment Unit–Medical University of Warsaw and Dept of Paediatrics of Children’s Hospital in Warsaw Previous medical history: respiratory, cardiovascular or neurological symptoms never arisen previously Nearly 72 h, discharged home	[12]
#**2** 17, M	*Emergency/* *non-fatal*	Legal high preparation, named HEX-EN	Intranasal	CNS alterations, i.e., psychomotor inhibition, psychosis, aggressiveness, bradycardia, hypotension, bradypnea, minimal Paediatric GCS point: 8 Muscle stiffness, CPK >, ALT >, AST	Symptomatic treatment	Legal high preparation: 3-CMC, HEX-EN (n-ethylhexedrone), MAB-CHMINACA, and caffeine	Admitted to Dept of Paediatrics and Clinical assessment Unit–Medical University of Warsaw and Dept of Paediatrics of Children’s Hospital in Warsaw Previous medical history: respiratory, cardiovascular or neurological symptoms never arisen previously Nearly 72 h, discharged home	[12]
#**3** 16, M	*Emergency/* *non-fatal*	Legal high preparation, named HEX-EN	Intranasal	CNS alterations, i.e., psychomotor inhibition, psychosis, aggressiveness (requiring physical restraint), bradycardia, hypotension, minimal Paediatric GCS point: 10. Muscle stiffness, CPK >, ALT, > AST	Symptomatic treatment	Legal high preparation: 3-CMC, HEX-EN (n-ethylhexedrone), MAB-CHMINACA, and caffeine	Admitted to Dept of Paediatrics and Clinical assessment Unit–Medical University of Warsaw and Dept of Paediatrics of Children’s Hospital in Warsaw Previous medical history: respiratory, cardiovascular or neurological symptoms never arisen previously About 9 days, discharged home	[12]
#**4** 20, M	*Emergency/* *non-fatal*	THC + unknown substance	smoke	CNS alterations, i.e., psychomotor agitation, anxiety, psychosis, aggressiveness/combativeness (requiring physical restrain), alternated with reduced consciousness (GCS: 4-6-1), tachycardia. Secondly: severe myoclonus, respiratory depression, urine retention	Symptomatic treatment including supplemental oxygen therapy, Sedation-Benzodiazepines (midazolam, iv)	Urine data: CMC + THC + Oppiates Powders content: caffeine and CMC	Taken to ED by ambulance staff, unknown medical history Caught in possession of 3 substances, i.e., cannabis, and 2 white powders labeled as “*hexen*” (n-ethylhexedrone) and “*speed*” Diagnosed for excited delirium syndrome (lasting over 24 h) due to withdrawal of drug of abuse nearly 48 h, discharged home	[11]
#**5** 20, M	*Emergency/* *non-fatal*	THC + unknown substance	smoke	CNS alterations (psychomotor agitation, anxiety, aggressiveness, psychosis), alternated with reduced consciousness/lethargy (GCS: 3-6-5), tachycardia Secondly: myoclonus, trismus, urine retention	Symptomatic treatment, Sedation-Benzodiazepines (midazolam and morphine iv)	Urine data: THC Powders content: caffeine and CMC	Taken to ED by ambulance staff, unknown medical history Caught in possession of 3 substances, i.e., cannabis, and 2 white powders labeled as “*hexen*” (n-ethylhexedrone) and “*speed*” Diagnosed for excited delirium syndrome (lasting over 24 h) due to withdrawal of drug of abuse Nearly 48 h, discharged home	[11]
#**6** 25, M	*Emergency/* *non-fatal*	AMP + unspecified NPS	−	CNS alterations, i.e., psychomotor agitation	−	Blood data: MMB-FUBINACA, AMP, BZO Urine data: AMP, tramadol, 3-CMC, BZO	Diagnosed for psychotic/behavioral disorders due to NPS abuse and withdrawal syndrome (F19.3, according to ICD-10) Unknown hospital stay duration	[13]
#**7** 24, F	*Emergency/* *non-fatal*	AMP	−	CNS alterations, i.e., fear, anxiety	−	Blood data: MMB-FUBINACA, AMP, BZO Urine data: AMP, tramadol, 3-CMC	Diagnosed for psychotic/behavioral disorders due to NPS abuse and withdrawal syndrome (F19.3, according to ICD-10) Unknown hospital stay duration	[13]
#**8**	*Non-fatal (self-reported)*	3-CMC	−	CNS alterations, i.e., long-lasting limbs paraesthesia and long-lasting pain, short term neurological adverse effects (possibly, Parsonage-Turner Syndrome)	Acetaminophen and codeine (self-administered)	−	Psychonaut–self reporting usual use of 3-CMC and other NPS	[14]
*Part B*								
*Case n./Sex, Age*	*Outcome*	*Case history, Anamnestic data, Annotations*		*Δ time from autopsy to toxicological examination of biological specimens*	*Analytical results* *in Blood*	*Analytical results* *in Urine*	*Polyabuse/co-assumed substances*	*References*
#**1** 29, F	*Fatal*	Found deceased after returning from a party. A bright-yellow substance was discovered spread in bedclothes		2 months	3-CMC	−	αPVT + αPHPP + αPHP	[15]
#**2** 44, M	*Fatal*	Known drug and alcohol user, found dead on stairs under a store		3 months	3-CMC	3-CMC	Ethanol	[15]
#**3** 22, F	*Fatal*	Found dead from drowning		5 months	3-CMC	3-CMC	Acetaminophen + Ethanol	[15]
#**4** 24, M	*Fatal*	Diagnosed for mental disorder; dead, fallen through a window		2 months	3-CMC	−	Thiopental+ Pentobarbital+ Oxycodone+ 4AA+ 4MAA+ 4FAA+ Acetaminophen	[15]
#**5** 48, M	*Fatal*	Dead after assumption of a mephedrone solution during a social gathering		1 months	3-CMC	3-CMC	−	[15]
#**6** 23, M	*Fatal*	Dead from cardiac arrest following a stabbing during a birthday party		3 months	−	3-CMC	Lidocaine + Ethanol	[15]
#**7** 42, M	*Fatal*	Discovered deceased in a church apartment; an intravenous cannula was found on the body inserted near the ankle		1 day after autopsy	3-CMC	3-CMC	−	[15]
#**8** 23, M	*Fatal*	Dead from cardiac arrest, the body found in a friend’s residence		5 months	−	3-CMC	MDA + MDMA + AMP + Ethanol	[15]
#**9** 25, M	*Fatal*	The body discovered close to the railroad bank and the river		5 months	−	3-CMC	COC + BZE + EME	[15]
#**10** 26, M	*Fatal*	Known psychiatric under treatment, suffering from neuralgia, drug addiction, illegal substances user. Dead after attempted hanging, ineffective cardiopulmonary resuscitation.		5 months	−	3-CMC	MDA + MDMA + BUP + clonazepam + 7-AC + lamotrigine + amiodarone + mirtazapine + atropine	[15]
#**11** 44, M	*Fatal*	Dead from hanging, the corpse found in a hotel room		4 months	−	3-CMC	Sildenafil	[15]
#**12** 29, M	*Fatal*	Dead in a motorcycle accident		7 months	−	3-CMC	−	[15]
#**13** 25, M	*Fatal*	Known drug addiction. Found deceased with a syringe close to the body. It turned out that he had a quarrel with his mother few hours before death.		4 months	−	3-CMC	Alprazolam + Tramadol	[15]

-: information missing; GCS: *Glasgow Coma Scale*; HEX-EN: *n-ethylhexedrone*; 3-CMC: *3-Chloromethcathinone*; MAB-CHMINACA: ADB- CHMINACA, *N-(1-amino-3,3-dimethyl-1-oxobutan-2-yl)-1-(cyclohexylmethyl)-1H-indazole-3-carboxamide*; CMC: *Chloromethcathinone*; THC: *cannabis*; ICD-10: *International Classification of Diseases (Psychiatric nosology), 10th edition (ICD-10), produced by the World Health Organization (WHO)*; AMP: *Amphetamines*; BZO: *Benzodiazepines*; MMB-FUBINACA: *methyl (1-(4-fluorobenzyl)-1H-indazole-3-carbonyl)valinate*; −: substance unidentified in the tested biological specimen; α-PVT: *α-pyrrolidinopentiothiophenone*; α-PHPP: *α-pyrrolidinoheptaphenone*; α-PHP: *α-pyrrolidinohexiophenone*; 4-AA: *4-aminoantipyrine*; 7-AC: *7-aminoclonazepam*; 4-FAA: *4-formylaminoantipyrine*; 4-MAA: *4-methylaminoantipyrine*; MDA: *3,4-methylenedioxyamphetamine*; MDMA: *(3,4-methylenedioxymethamphetamine)*; BZE: *benzoylecgonine*; EME: *ecgonine methyl ester*; BUP: *buprenorphine* (modified from [15], describing deaths reported at the Department of Forensic Medicine in Kraków between 2016 and 2022).

## 2. Results

### 2.1. Breath Rate

**Figure 2:**
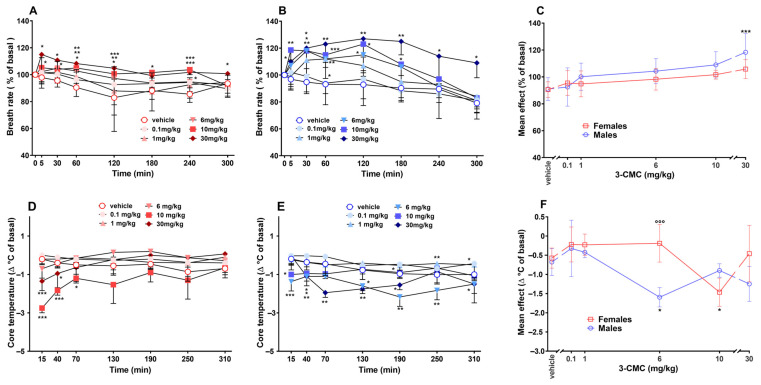
Effect of 3-CMC (0.1–30 mg/kg; i.p.) on breath rate [in female (**A**) and male (**B**) mice] and on core temperature [in female (**D**) and male (**E**) mice], and comparison of main effect of each treatment (**C**,**F**). Data are expressed as percentage of basal values (**A**–**C**) and as difference (Δ°C) with respect to basal values (**D**–**F**; Mean ± SD, *n* = 6/group). Statistical analysis was performed by Two-way RM ANOVA followed by Bonferroni’s test for multiple comparisons (**A**,**B**,**D**,**E**) and by Two-way ANOVA followed by Bonferroni’s test for multiple comparisons (**C**,**F**). * *p* < 0.05; ** *p* < 0.01, *** *p* < 0.001 versus vehicle; °°° *p* < 0.001 versus male group.

The breath rate of vehicle groups was stable during the experiment. In female mice [Figure 2A; Two-way RM ANOVA, Table S1], the systemic administration of 6 (from 60 to 120 min post-injection), 10 (from 5 to 240 min post-injection), and 30 (at all time points except for 180 min post-injection) mg/kg of 3-CMC caused a mild increase in the frequency. Moreover, Two-way RM ANOVA detected a significant effect for the lowest dose tested (0.1 mg/kg) at 240 min post-treatment. A similar effect was observed in male mice treated with 1 (at 60 min post-injection), 6 (from 30 min to 120 min), 10 (from 5 min to 180 min), and 30 (from 5 min to the end of the test) mg/kg [Figure 2B; Two-way RM ANOVA, Table S1]. A significant main effect was calculated only for the male group treated with the highest dose [Figure 2C; Two-way ANOVA, main effect of sex F(1,60) = 4.461 and *p* = 0.0389; dose F(5,60) = 8.002 and *p* < 0.0001, non-significant sex × dose interaction F(5,60) = 0.9704 and *p* = 0.4432].

### 2.2. Body Temperature

The core temperature of vehicle-treated mice remained stable during the overall time of the experiment. The systemic administration of 3-CMC caused an immediate but transient decrease in body temperature of female mice at the doses of 10 (from 15 to 50 min) and 30 (from 15 to 40 min) mg/kg [Figure 2D; Two-way RM ANOVA, Table S1]. In males, a statistical significance was detected for the doses of 6, 10, and 30 mg/kg, which caused a small but more prolonged hypothermia. On the other hand, the analysis detected a significant increase in temperature in mice treated with 1 mg/kg from 190 min to the end of the experiment [Figure 2E; Two-way RM ANOVA, Table S1]. 3-CMC induced a significant mean effect at the doses of 6 (in males only) and 10 (in females only) mg/kg. A significant difference between the two groups treated with 6 mg/kg was revealed [Figure 2F; Two-way ANOVA, main effect of sex F(1,60) = 10.98 and *p* = 0.0016; dose F(5,60) = 7.928 and *p* < 0.0001, sex × dose interaction F(5,60) = 7.298 and *p* < 0.0001].

### 2.3. Visual Responses

**Figure 3:**
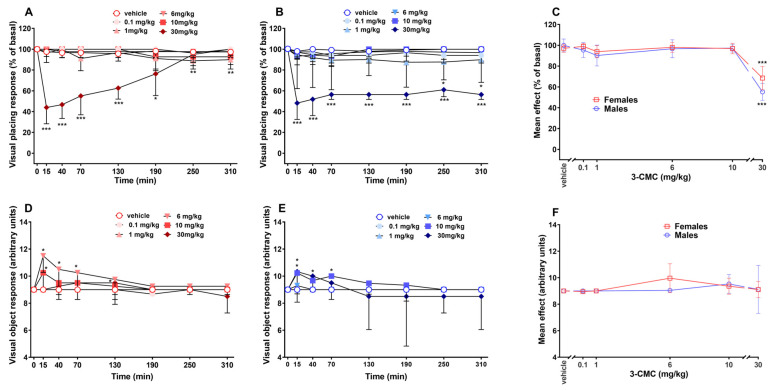
Effect of 3-CMC (0.1–30 mg/kg; i.p.) on visual placing [in female (**A**) and male (**B**) mice] and visual object [in female (**D**) and male (**E**) mice] response, and comparison of main effect of each treatment (**C**,**F**). Data are expressed as percentage of basal values (**A**–**C**) and as arbitrary units (**D**–**F**; Mean ± SD, *n* = 6/group). Statistical analysis was performed by Two-way RM ANOVA followed by Bonferroni’s test for multiple comparisons (**A**,**B**,**D**,**E**) and by Two-way ANOVA followed by Bonferroni’s test for multiple comparisons (**C**,**F**). * *p* < 0.05; ** *p* < 0.01, *** *p* < 0.001 versus vehicle.

The visual placing did not change in the vehicle groups. 3-CMC resulted to induce an impairment of the visual response only at the highest dose tested in both sexes: in females, the effect was transient [Figure 3A; Two-way RM ANOVA, Table S1], while in males it was long-lasting [Figure 3B; Two-way RM ANOVA, Table S1]. Moreover, a significance was detected for the lowest dose (0.1 mg/kg) and the second lowest dose (1 mg/kg) tested in female and male mice, respectively, at 250 and 310 min post-treatment (Figure 3A,B). In both sexes, 3-CMC induced a significant mean effect only with the highest dose tested [Figure 3C; Two-way ANOVA, non-significant effect of sex F(1,60) = 1.962 and *p* = 0.1665; significant effect of dose F(5,60) = 27.38 and *p* < 0.0001, non-significant sex × dose interaction F(5,60) = 1.035 and *p* = 0.4052].

The visual object response did not change in the vehicle groups. The injection of 3-CMC induced a transient but significant enhancement of the response in females (Figure 3D), but only at the doses of 6 (until 70 min) and 10 (only at 15 min) mg/kg [Two-way RM ANOVA, Table S1]. In males, the analysis resulted in a non-significant effect of treatment [Two-way RM ANOVA, Table S1], but post hoc test revealed a significance for the doses of 6 (only at 15 min), 10 (only at 70 min), and 30 (between 40 and 70 min) mg/kg (Figure 3E). 3-CMC did not induce a relevant mean effect in either male or female mice (Figure 3F).

### 2.4. Acoustic Responses

**Figure 4:**
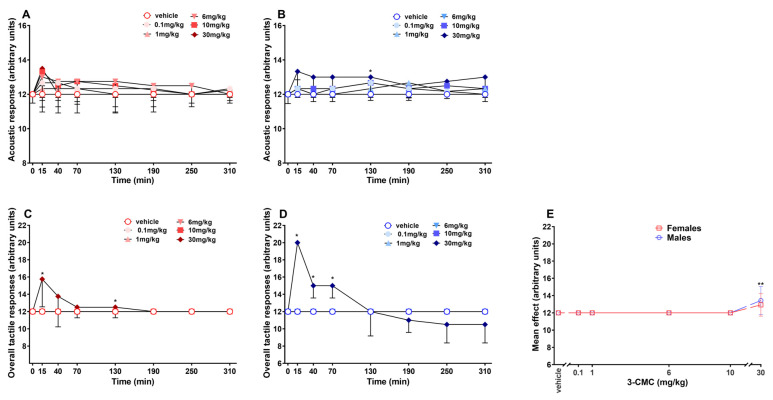
Effect of 3-CMC (0.1–30 mg/kg; i.p.) on acoustic [in female (**A**) and male (**B**) mice] and tactile [in female (**D**) and male (**E**) mice] response, and comparison of main effect of each treatment (**E**). Data are expressed as arbitrary units (Mean ± SD, *n* = 6/group). Statistical analysis was performed by Two-way RM ANOVA followed by Bonferroni’s test for multiple comparisons (**A**,**B**,C,**D**) and by Two-way ANOVA followed by Bonferroni’s test for multiple comparisons (E). * *p* < 0.05; ** *p* < 0.01 versus vehicle.

In vehicle groups, no change in the acoustic reflex was observed. The systemic administration of 3-CMC did not induce relevant effects in female mice [Figure 4A; Two-way RM ANOVA, Table S1]. Differently, in males, a small increase in the acoustic reflex was observed in the 30 mg/kg group, only at 130 min post-injection [Figure 4B; Two-way RM ANOVA, Table S1].

### 2.5. Tactile Responses

The tactile reflex of vehicle-treated mice did not change during the experimental session. Systemic administration of 3-CMC induced changes in the response of both sexes, but only at the highest dose (30 mg/kg). Specifically, in females, a significant increase in the reflex was observed at 15 and again at 130 min after the treatment [Figure 4C; Two-way RM ANOVA, Table S1]. In males, the same dose caused a relevant alteration until 70 min post-treatment [Figure 4D; Two-way RM ANOVA, Table S1]. The statistical analysis of the mean effects revealed a significant increase in tactile response only in male mice treated with the highest dose tested [Figure 4E; Two-way ANOVA, non-significant effect of sex F(1,60) = 0.3451 and *p* = 0.5591, significant effect of dose F(5,60) = 7.669 and *p* < 0.0001, non-significant sex × dose interaction F(5,60) = 0.3451 and *p* = 0.8834].

### 2.6. Locomotory Activity

**Figure 5:**
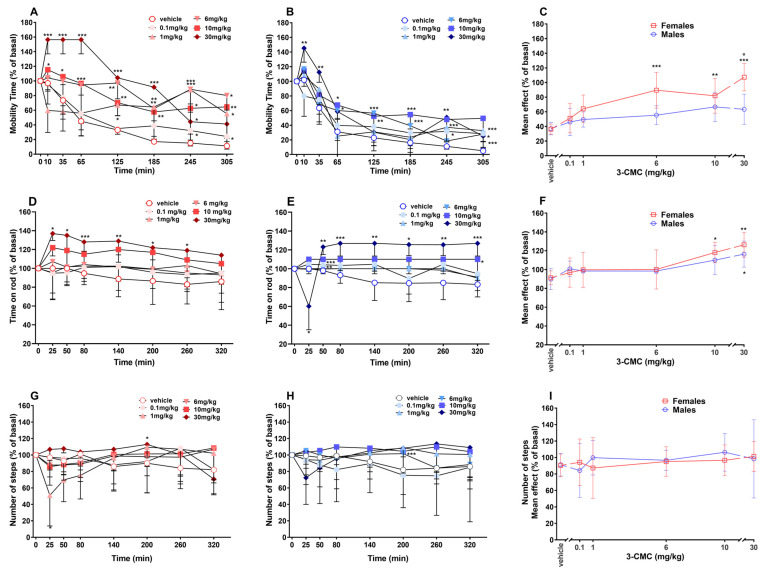
Effect of 3-CMC (0.1–30 mg/kg; i.p.) on mobility time [in female (**A**) and male (**B**) mice], time of rotarod [in female (**D**) and male (**E**) mice], number of steps [in female (**G**) and male (**H**) mice] and comparison of main effect of each treatment (**C**,**F**,**I**). Data are expressed as a percentage of basal values (Mean ± SD, *n* = 6/group). Statistical analysis was performed by Two-way RM ANOVA followed by Bonferroni’s test for multiple comparisons (**A**,**B**,**D**,**E**,**G**,**H**) and by Two-way ANOVA followed by Bonferroni’s test for multiple comparisons (**C**,**F**, **I**). * *p* < 0.05; ** *p* < 0.01; *** *p* < 0.001 versus vehicle; ° *p* < 0.05 versus male group.

The spontaneous locomotion of mice treated with the vehicle progressively reduced during the experiment. The systemic administration of 3-CMC increased the mobility time of both groups. In females, the effect was observed at all dosages. The effect of 10 and 30 mg/kg doses was immediate, while the other doses induced a retarded effect [Figure 5A; Two-way RM ANOVA, Table S1]. On the other hand, 3-CMC seemed to induce less pronounced effects in male mice, for which significance was detected for the doses of 30 (until 35 min and again after 245 min), 10 and 6 (between 65 and 185 min), 1 and 0.1 (after 245 min to the end of the experiment) mg/kg [Figure 5B; Two-way RM ANOVA, Table S1]. 3-CMC induced a significant mean effect only in females starting from the dose of 6 mg/kg. A significant difference was also detected between the two sexes at the 30 mg/kg dose [Figure 5C; Two-way ANOVA, main effect of sex F(1,60) = 17.63 and *p* < 0.0001; dose F(5,60) = 11.11 and *p* < 0.0001, sex × dose interaction F(5,60) = 2.461 and *p* = 0.0429].

The injection of the vehicle did not affect the stimulated motor activity of mice. 3-CMC enhanced the time spent on the rotarod of female mice only at doses of 30 mg/kg [Figure 5D; Two-way RM ANOVA, Table S1]. A similar effect was observed in male mice, despite the highest dose (30 mg/kg) initially caused a reduction in the time spent on the rotarod, followed by a prolonged increase in the performance [Figure 5E; Two-way RM ANOVA, Table S1]. Moreover, it resulted in a transient but significant effect for the doses of 1 and 6 mg/kg (only at 50 min) and 10 mg/kg (only at 320 min).

A relevant mean effect was induced by the treatment with 10 (in females only) and 30 (in both sexes) mg/kg [Figure 5F; Two-way ANOVA, non-significant effect of sex F(1,60) = 1.003 and *p* = 0.3205; significant effect of dose F(5,60) = 9.426 and *p* < 0.0001, non-significant sex × dose interaction F(5,60) = 0.4699 and *p* = 0.7972].

Vehicle-treated mice exhibited no variation in the number of steps. Systemic administration of 3-CMC did not affect the response of female mice, except for the dose of 1 mg/kg, which induced a transient decrease in the number of steps, and the dose of 30 mg/kg, which caused a significant increase at 200 min [Figure 5G; Two-way RM ANOVA, Table S1]. For the male group, the compound did not induce relevant effects, except for the 6 mg/kg dose at 200 min post-treatment [Figure 5H, Two-way RM ANOVA, Table S1]. The Two-way ANOVA analyses did not detect significant mean effect in the two sexes (Figure 5I).

### 2.7. PPI Test

**Figure 6:**
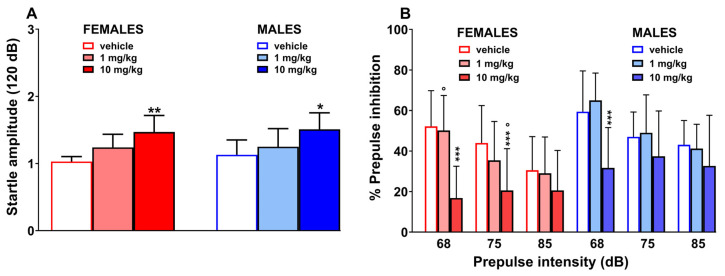
Effect of 3-CMC (1 and 10 mg/kg; i.p.) on the startle (**A**) and PPI (**B**) response in female and male mice. Data are expressed as absolute values (**A**) and as percentages of basal values (**B**); Mean ± SD, *n* = 6/group). Statistical analysis was performed by One-way ANOVA followed by Bonferroni’s test for multiple comparisons (3-CMC versus vehicle; (**A**), by Student’s t test to compare the effects in the two sexes (**A**), and by Three-way ANOVA followed by Bonferroni’s test for multiple comparisons (**B**). * *p* < 0.05, ** *p* < 0.01, *** *p* < 0.001 versus vehicle; ° *p* < 0.05 versus male group.

The systemic administration of 10 mg/kg of 3-CMC in the female group induced an increase in the startle response [Figure 6A; One-way ANOVA, F(2,15) = 8.399 and *p* = 0.0036) and a similar effect was observed in the male group [Figure 6A; One-way ANOVA, F(2,15) = 3.748 and *p* = 0.0478]. Based on the Three-way ANOVA analysis, it resulted that the dose of 1 mg/kg does not induce significant effect either in males or in females with respect to vehicle, but a difference was detected between the two sexes at the prepulse intensity of 68 dB [Figure 6B; Three-way ANOVA; significant effect of prepulse intensity F(2,60) = 57.44 and *p* < 0.0001; non-significant effect of treatment F(1,60) = 0.4571 and *p* = 0.5016; significant effect of sex F(1, 60) = 44.09 and *p* < 0.0001; non-significant effect of prepulse intensity × treatment F(2,60) = 0.8822 and *p* = 0.4192, prepulse intenxity × sex F(2,60) = 0.5596 and *p* = 0.5744; treatment × sex F(1,60) = 3.494 and *p* = 0.0665; prepulse intensity × treatment × sex F(2,60) = 1.052 and *p* = 0.3555]. On the other hand, the high dose induced a relevant effect in both sexes at 68 dB, while only in females at 75 dB. Finally, a difference was detected between the %PPI of females and males at 75 dB [Three-way ANOVA; significant effect of prepulse intensity F(2,60) = 7.342 and *p* = 0.0014; treatment F(1,60) = 116.2 and *p* < 0.0001; sex F(1, 60) = 37.94 and *p* < 0.0001; prepulse intensity × treatment F(2,60) = 12.33 and *p* < 0.0001, prepulse intensity × sex F(2,60) = 0.1447 and *p* = 0.8656; treatment × sex F(1,60) = 3.762 and *p* = 0.0571; prepulse intensity × treatment × sex F(2,60) = 1.319 and *p* = 0.2750].

### 2.8. Human Intoxication Cases

Over the 11-year evaluated period (2014–2025), a total of three identified 3-CMC abuse cases were documented, with another two recorded cases involving CMC, for which the identification/distinction between isomers (3-CMC vs. 4-CMC) was not achievable (Table 2). All these five poisonings occurred in the two-year period 2022/2023, starting from January 2022. Notably, any intoxication was chronicled neither between 2014 and 2021, nor between 2024 and 2025.

CMC was consumed alone or in association with other substances, e.g., alkyl nitrite inhalants (“popper”), or NPS. Users were exclusively men (5/5), with a mean age of 34.2 years (23/43 years old), self-reporting occasional (2/7) or habitual (1/7) NPS consumption in a sexual gay context (5/5) through different routes of exposure, i.e., nasal, sniffing or snorting (2/5), oral (2/5), and/or smoking (1/5). The injection route, both intravenous and intramuscular, was also reported (1/5). CMC tended to be experienced by polydrug users (4/5); in fact, almost all of the patients reported use of cocaine, methadone, or other drugs such as GHB and 4-MMC (mephedrone), contextually or even recurrently. Moreover, some patients presented alcohol dependence (1/5) or contextual alcohol intake (1/5).

The exclusive assumption of CMC, orally ingested, was determined in only one case (1/5). Notably, no patients intentionally consumed CMC (3-CMC or 4-CMC isomers), thus consuming this NPS unaware; in fact, 4/5 subjects referred to cathinones (e.g., mephedrone, 3-methylmethcathinone, α-PVP) consumption, and 1/5 reported GHB intake, but confirmatory analyses assessed the presence of CMC, and in most cases, 3-CMC identification was achieved.

As previously reported for other cathinones [16,17], in the present clinical series, CMC was consumed in a sexual gay context (5/5), among men who have sex with men (MSM), namely ChemSex. ChemSex, also known as Slamsex, indicates the use of certain substances before or during sexual interaction to improve and prolong performance. The Slamsex phenomenon has attracted attention worldwide, causing great public health concern, since it is often associated with a high prevalence of HIV/HCV infection, even though this dangerous link has not been recorded in our current cases.

Relevant toxidromes were described in all five patients testing positive for CMC, or specifically for 3-CMC, even though all recorded cases were intentional, non-fatal intoxications. The clinical manifestations included both central and peripheral symptoms; specifically, neurological/psychiatric and respiratory symptoms were reported. Cardiotoxicity was also recognized. Almost all cases presented behavioral/neuropsychiatric symptoms, including psychomotor agitation (4/5), mental confusion/psychosis (4/5), or, contrarily, CNS depression and coma (1/5). One patient experienced auditory and visual hallucinations (1/5). Respiratory distress (including tachypnoea) and desaturation (88% in ambient air), paralleled by 14% methemoglobinemia, were observed in only one case, which tested positive for 3-CMC and alkyl nitrites.

An aggressive, psychotic self-injuring behavior, resulting in a suicide attempt, was registered in one of the two cases positive for CMC, but for which the distinction between CMC isomers (3-CMC vs. 4-CMC) was not achievable. Three cases presented signs of potential cardiotoxicity, including cardiac arrhythmias (e.g., tachycardia and extrasystole) and thoracic pain, all confirmed positive for 3-CMC. Other peripheral symptoms were observed in single cases, e.g., mydriasis, miosis, and vomiting.

Out of the total, some subjects (3/5) were taken to EDs by police/ambulance staff, due to their aggressive behavior or psychomotor agitation condition, whereas in the other cases, patients moved to EDs by self-referral.

For the patients’ management, in light of the crucial need for a rapid treatment of psychomotor agitation in all patients, symptomatic treatment and supportive care remained the mainstay. Pharmacological sedation using benzodiazepines (e.g., midazolam) and neuroleptic/antipsychotic drugs, e.g., clotiapine and promazine, was documented in some cases (3/5). Also, hydration was reported in almost all cases. After healings, clinical signs/symptoms resolution arose quite rapidly, with patients discharged home (4/5) or transferred to psychiatric ward (1/5) within 30 h after hospital admission. Antidotal treatment, using methylene blue, was administered only in one case, to treat alkyl nitrites-induced methemoglobinemia.

### 2.9. ADMET Predictions

For CMCs (Figure S1), the simulator predicted N-dealkylation (M2), N-hydroxylation (M3), in addition to oxidative deamination (3-CMC-M6, 2-CMC-M6, and 4-CMC-M5). Phenyl hydroxylation was predicted only for 2-CMC (at position 4 or 5, 2-CMC-M4) and 3-CMC (at position 4, 3-CMC-M4). Similarly, the main predicted metabolites of 2-MMC (Figure S2) resulted from N-dealkylation (M2), phenyl hydroxylation at position 4 (M3), and hydroxylation at the benzylic position (M4). Likewise, oxidative deamination was predicted for 2-MMC (M6).

Table 3 shows the cytochromes (CYPs) predicted to be involved in the metabolism of the tested drugs and the potential of compounds to inhibit them. It was found that all CMCs are substrates for CYP1A2, CYP2A6, CYP2C8, CYP2C19, CYP2D6, and CYP2E1. 2-MMC was predicted to be a substrate for the same CYPs, except for CYP2C8. Both metabolites of 3-CMC were predicted to be substrates for CYP2E1 and CYP2C19. CYP1A2 and CYP2A6 were predicted for N-demethyl-3-CMC, while CYP2C8 and CYP2D6 were predicted for dihydro-3-CMC. All tested compounds, except for 2-MMC and dihydro-3-CMC, were shown to inhibit CYP1A2.

As shown in Table 4, all tested compounds were predicted to have a high permeability and to highly permeate across the BBB (blood–brain barrier). None of the compounds was shown to inhibit the P-glycoprotein (PgP); however, 2-MMC and dihydro-3-CMC were predicted to be substrates for PgP and organic cation transporter 2 (OCT2). The inhibition of OCT2 was predicted for all tested compounds. The only compound predicted to interact with organic cation transporter 1 (OCT1, both a substrate and an inhibitor) is dihydro-3-CMC. The calculated volume of distribution (Vd) indicates high distribution at steady state for all compounds.

Table 5 shows that none of the compounds was predicted to induce high levels of serum alkaline phosphatase (Ser_AlkPhos). The only compound predicted to increase serum levels of Gamma-glutamyl transferase (Ser_GGT) is 2-MMC. The induction of elevated levels of serum lactate dehydrogenase (Ser_LDH) was predicted for all compounds. Elevation of aspartic acid transaminase (Ser_AST) was predicted for 3-CMC, 2-CMC, and the two metabolites. Elevation of alanine transaminase (Ser_ALT) was predicted for 3-CMC, 2-CMC, and N-demethyl-3-CMC. The predicted intrinsic clearance in human hepatocytes (HEP hCLint) of all compounds is within the recommended range (>60.90) and was higher for CMCs. All compounds were shown to block the hERG potassium channel and have similar affinity.

## 3. Discussion

In the current work, we present the physiological and sensorimotor effects in female and male mice of a recently increasing circulating synthetic cathinone: 3-CMC, one of the most seized compounds in Europe [1,2], also associated with intoxication cases in Italian territory. Thus, we report a case series involving 3-CMC (or CMC) that occurred in an 11-year period. Since the identification of the specific isomer is not always possible, we also conducted an in silico study to determine the ADMET profile and metabolic pathway of 3-CMC and its structural isomers (4-CMC and 2-CMC), in addition to 2-MMC. Even this latter cathinone, for which a paucity of data exists, with only one published in vitro study [18], could re-emerge as a replacement for the controlled analogues [7]. Finally, we studied the ADMET profile of two 3-CMC metabolites in order to evaluate their toxicity.

### 3.1. Effects of 3-CMC in Mice

As previously observed with other stimulants and SCs [16,19], 3-CMC injection increased the breath rate of both female and male mice. This effect is in line with the mechanism of action of 3-CMC, which induces the release of NE [10]. Indeed, it is known that noradrenergic system takes part in the neuromodulation of respiratory rhythm, acting through α1- and α2-receptors and modulating pacemaker and non-pacemaker neurons [19,20,21]. Sex seems to have an impact on this effect, as it was observed at a lower dose in male mice, suggesting a higher sensitivity. Currently, there are no studies evaluating the effect of SCs on this parameter in female rodents; hence, more detailed studies should be conducted to assess the cardiorespiratory effects of this compound in both sexes.

The systemic administration of 3-CMC in mice also induced a reduction of the body core temperature, aligning with its isomer 4-CMC [22] and mephedrone [23,24], for which the role of serotonergic system has been postulated. In previous studies [16,19] conducted on psychostimulants with a more dopaminergic profile of action, a similar hypothermia was observed, which suggests a mechanism involving dopamine. Indeed, it has been shown that dopamine receptor activation induces hypothermia in rodents [25]. On the other hand, Shortall et al. showed that the hypothermic effect of mephedrone was attenuated (but not fully blocked) by the depletion of 5-TH and by a 5-HT1B antagonist [24]. Hence, it is not clear how 3-CMC induces this mild alteration, but it is worth noting that, contrary to the observed dose-dependent hypothermic effect of other SCs [26], the doses of 6 (in males) and 10 (in females) mg/kg induced a more relevant reduction in core temperature with respect to 30 mg/kg. Hence, it’s worth exploring the mechanism involved in this effect of 3-CMC, the reasons behind the observed sex differences, and if a further dose increase could evolve in hyperthermia.

In line with other psychostimulants [16,19], 3-CMC induced (only at the highest dose tested) an impairment of the visual placing response of both female and male mice, suggesting an alteration of the cerebellar synaptic functions and the activation of the noradrenergic system. Indeed, NE modulates the vestibulo-ocular reflex and the optokinetic response through the activation of β-adrenergic receptor (at low concentrations) and α2-receptor (at high concentrations) [27]. On the other hand, treated mice tended to exert higher responses to visual objects, acoustic (only males), and tactile stimulations. It is not clear the mechanism behind these effects; however, Zhou and colleagues found that methcathinone enhanced the neuronal activity in the motor cortex, somatosensory cortex, visual cortex, and piriform area, and improved the coding efficiency and precision of visual cortical neurons. The authors suggested the activation of neurons expressing D1-receptors and the inhibition of those expressing D2-receptors [28]. Hence, it is possible that 3-CMC induces similar effects in mice, but more detailed studies should be conducted to confirm this hypothesis.

As expected, among the effects of 3-CMC in mice, the increase in the spontaneous locomotor activity was the predominant one. In fact, 3-CMC interacts with DAT (EC50 = 46.8 ± 4.0 nM) [10] and increases DA transmission, which is known to be the basis for motor stimulation induced by psychostimulants [29,30,31]. Females appeared to be more sensitive to this effect, as previously described with other compounds [32,33,34]. The dose of 1 mg/kg induced in females an initial and transient decrease in the number of steps at the drag test. The mechanism behind this is not clear; however, 3-CMC also interacts with SERT (EC50 = 410 ± 38 nM) [10] and induces the release of 5-HT. Hence, as proposed for a different SC [17], it is possible that low doses of 3-CMC trigger the action of 5-HT receptors, shown to be implicated in the inhibition of locomotory activity induced by the psychedelic phenethylamine DOI [35,36]. High doses of 3-CMC also induced a facilitation of locomotion at the rotarod test in both groups, contrasting with the study of Wojcieszak and colleagues [37], in which the compound (5, 10, and 20 mg/kg; s.c.) did not affect the performance of mice. However, differences in the protocol could explain this apparent discrepancy. The effect was quite similar between the two sexes; however, a transitory impairment of motor coordination was observed in males after the injection of 30 mg/kg, explainable with the appearance of stereotypies [38,39,40].

To test the effect on sensorimotor gating [41], we conducted the Startle/PPI test. The obtained data showed that 10 mg/kg increased the startle amplitude and inhibited the PPI in both female and male mice, despite females resulted to be more sensitive to this effect. The dopaminergic system is possibly involved in this effect, as suggested by previous studies with MDPV and cathinone [42,43]. However, based on the selectivity towards DAT versus SERT of 3-CMC (about 8-fold) [10], we cannot exclude a serotonergic mechanism, commonly linked to PPI and sensorimotor alterations [44]. Additionally, mephedrone (4-MMC), methcathinone, and flephedrone (4-FMC) interact with 5-HT2A [45]. Thus, it is possible that 3-CMC induces alterations in PPI through the action, both direct and indirect (i.e., via SERT), on serotonin receptors.

### 3.2. Human Poisonings

In the current paper, we report CMC-human intentional non-fatal intoxication cases in subjects admitted to EDs, inside the NEWS-D Project, from January 2014 to February 2025, managed by the PCC. Notably, our data documented that, over the 11-year evaluated period, poisonings were recorded in the two-year period 2022–2023, starting from January 2022, with three analytically confirmed 3-CMC abuse cases and another two for which the distinction between isomers, i.e., 3-CMC vs. 4-CMC, was not attainable.

Intoxications involved only male subjects, who always experienced CMC in a sexual gay context, associated with other NPS/substance consumption or even alcohol intake. Remarkably, any case involving CMC was documented neither in the period between 2014 and 2021 nor in 2024–2025.

Actually, the drug market is dramatically rising worldwide, constantly proposing newly synthetized substances, despite others declining over time, then possibly returning to be used [6,46,47,48,49]. Hence, based on our data reporting CMC abuse exclusively during 2022–2023, we could realistically assume the existence of fluctuation in the NPS consumer trends, in which 3-CMC could have emerged to replace the “older” 4-CMC, just banned at that time [1,6,7,50], then in turn be replaced by other substances.

Notably, the reported intoxications demonstrate the pervasiveness of ChemSex phenomenon, highlighting the MSM’s voluntary intake of synthetic cathinones (SCs) in recreational settings, as sexually disinhibiting substances with the aim of improving sexual performance [51,52,53,54].

In line with existing literature data listed in Table 1, in particular with Part A referring to CMC non-fatal intoxications, we documented representative toxidromes characterized by toxicity signs/symptoms: (i) neurological/behavioral/neuropsychiatric, including psychomotor agitation, psychosis, aggressiveness, or contrarily CNS depression, (ii) cardiovascular/respiratory, e.g., cardiac arrhythmias, tachypnoea, thoracic pain, and peripheral (alteration of pupillary condition, vomiting).

Another important matter to be highlighted is the incongruity existing between self-reporting anamnestic information and subsequent analytical findings; specifically, it has to be highlighted that NPS users do not always know correctly what they are consuming, since the real content of the products, almost exclusively gained online, can greatly vary from that specified on the packaging label. In fact, none of our recorded intoxicated subjects intend to use specifically CMC (or 3-CMC), hence consuming these substances completely unaware.

Moreover, as already reported for other NPS, CMC toxidromes are characterized by overlapping, unusual signs/symptoms, hence hindering clinicians’ ability to accurately identify the causative agent and to guide development of a prompt differential diagnosis. Currently, specific practice guidelines need to be developed for intoxicated patients. Patients must therefore be monitored closely, and evaluation of their status is imperative, with supportive treatment and symptom management still remaining the mainstay [55,56]. For this reason, in the emergency medicine setting, a great effort is continuously devoted to identifying valuable antidotal treatments to be used for these challenging patients, and a multidisciplinary approach, involving specialized PCC toxicologists, considering altogether clinical/analytical findings and patients’ medical history, is strongly endorsed.

As previously reported for other NPS poisonings, even for the current case reports study some limitations could be highlighted, e.g., missing data about dosing, exposure route/frequency of intake, unobtainable information regarding the consumption time (affecting the sampling time standardization), and urgent need for reference standards of parent drugs and/or metabolites, which may have hampered the analytical assessment of SCs/NPS. Further, it has to be noted that the number of our case reports is limited, i.e., five CMC poisoning occurring recently in a two-year time window, over a 11-year evaluated period, possibly due to changes in NPS market and relative consuming trends or even to some factors which might possibly have dissuaded the NPS abusers to request urgent care, such as legal consequences or underestimate the health outcomes. Finally, it has to be stated that this case series does not comprise NPS poisonings which have been diagnosed as conventional drug intoxications or as unidentified drug exposure at ED admission without consulting PCC toxicologists.

### 3.3. ADMET Predictions

Based on the in silico investigation, one of the predicted main metabolites of 3-CMC is N-demethyl-3-CMC, coherently with previous studies describing the identification of 3-CMC metabolites in both mice [57] and humans [15,58]. Similarly, 4-CMC was predicted to undergo N-demethylation, in line with the existing literature [59,60]. Thus, the ADMET Predictor confirms itself to be a useful in silico tool to make a rapid and valid prediction of new compounds, allowing for the determination of possible markers for the detection of NPS. Nonetheless, the program failed to predict the dihydro-3-CMC and dihydro-4-CMC, reported in the above-mentioned studies. Thus, in vitro and in vivo preclinical research still have a crucial role in the complete understanding of the metabolism of drugs, including NPS. Based on the similar pathways described for 2-CMC and 2-MMC, and on available literature on metabolism of CMCs [59] and MMCs [61,62], it is likely that both compounds undergo carbonyl reduction. Nevertheless, dihydro-2-MMC is not produced in pooled liver microsomes [18]; thus, in vivo investigation is required to confirm the hypothesis.

Similarly to cathinone [63], all tested CMCs were predicted as possible substrates and inhibitors (along with N-demethyl-3-CMC) for CYP1A2, while 2-MMC possibly interacts with CYP2D6 and CYP2E1. The tested compounds also resulted in interacting with PgP, OCT1, and OCT2, which can lead to altered pharmacokinetic and drug toxicity. All of these suggest potential drug–drug interactions between tested cathinones and other substances (including prescription drugs) [64,65]. Finally, coherently with their chemical structures, 3-CMC and its isomers have similar PK properties, including permeability, capability to cross the BBB, and Vd.

A hepatotoxic potential emerged in particular for 3-CMC, 2-CMC, and N-demethyl-3-CMC. Different SCs were previously found to induce hepatocellular toxicity and hepatic injuries [66,67,68]. Moreover, increased AST and ALT levels were reported for two patients after the consumption of a “legal high preparation” containing 3-CMC (Table 1). Interestingly, 4-CMC and 2-MMC were found to induce elevation of hepatic enzymes to a lesser extent, which suggests that the presence of the halogen in a particular position at the phenyl ring enhances toxicity. Additionally, all compounds (including 4-CMC and 2-MMC) were predicted to raise the serum LDH, an indicator of acute renal failure induced by rhabdomyolysis [69], which can be observed in the case of consumption of SCs [70,71].

At present, there is a lack of information on the half-life of the tested SCs; however, the results of a recent in vitro study [72] align with the low intrinsic clearance predicted for 3-CMC and 4-CMC. Finally, based on the hERG block prediction, all compounds seem to have a potential for cardiotoxicity, which aligns with the cardiovascular manifestation observed in reports of intoxications with SCs [73,74].

## 4. Materials and Methods

### 4.1. Animals

A total of 54 male and 54 female ICR (CD-1^®^) mice, 3–4 months old, weighing 25–30 gr (ENVIGO Harlan Italy, Correzzana, Italy; bred inside the Laboratory for Preclinical Research (LARP) of the University of Ferrara, Italy), were used for this study. Mice were group-housed (8 mice per cage; floor area per animal of 80 cm^2^; a minimum enclosure height of 12 cm) and were exposed to a 12:12-h light-dark cycle (light on at 6:30 AM), temperature of 20–22 °C, and humidity of 45–55%. Ad libitum access to food (Diet 4RF25 GLP; Mucedola, Settimo Milanese, Milan, Italy) and water was provided. The experimental protocols performed in the present study were in accordance with the U.K. Animals (Scientific Procedures) Act of 1986 and associated guidelines and the new European Communities Council Directive of September 2010 (2010/63/EU [75]). Experimental protocols were approved by the Italian Ministry of Health (license n. 223/2021-PR) and by the Animal Welfare Body of the University of Ferrara. According to the ARRIVE guidelines, all possible efforts were made to minimize the number of animals used, the pain and discomfort of the subjects. Twelve mice (6 males, 6 females) were used for each treatment: vehicle or 0.1 mg/kg, 1 mg/kg, 6 mg/kg, 10 mg/kg, and 30 mg/kg of 3-CMC (for behavioral study), for a total of 36 female and 36 male mice. Twelve mice (6 males, 6 females) were used for each treatment: vehicle or 1 mg/kg and 10 mg/kg of 3-CMC, for a total of 18 female and 18 male mice. GraphPad calculator was employed to randomly assign 6 animals/group. The sample size was calculated by applying the prior power analysis [76]. All experiments were performed between 8:30 and 2:00 PM.

### 4.2. Chemical and Reagents

3-CMC was purchased from LGC Standards (Milan, Italy) as the hydrochloride salt. The substance was dissolved in saline (0.9% p/v NaCl), which was also employed as a vehicle. 3-CMC was administered by the intraperitoneal route (volume of 4 µL/gr). Online reports of users [9] and the interspecies dose scaling [77] were used to select the drug doses: 0.1 and 1 mg/kg correspond to an under-threshold dose, 6 mg/kg to a light dose, 10 mg/kg to a common dose, and 30 mg/kg to a strong/heavy dose in humans.

### 4.3. Behavioral Study

The effects induced in mice by 3-CMC (0.1–30 mg/kg, i.p.) on behavioral responses were investigated through a battery of tests (Safety Pharmacology) routinely employed in our laboratory for the preclinical characterization of new molecules [78,79]. With the aim of reducing the number of mice employed, the sensorimotor response tests were conducted consecutively according to the following time scheme: breath rate, mobility time, visual (placing and object) responses, acoustic responses, overall tactile responses, body core temperature, time on rod, and number of steps. The test battery was conducted at 0 (prior to administration), 5, 30, 60, 120, 180, 240, and 300 min after the treatment. For more detailed information about time points, see Supplementary Materials (Table S2).

Experiments were carried out in a laboratory with controlled temperature (20–22 °C), humidity (45–55%), light (150 lux), and background noise (40 ± 4 dB). The behavioral tests were conducted by blinded and trained operators working in pairs and videotaped by a camera (B/W USB Camera day and night with varifocal lens; Ugo Basile, Italy) placed at the top or on one side of the box and analyzed offline by a different trained operator.

#### 4.3.1. Breath Rate

For the evaluation of breath rate, mice were placed individually in cages and allowed to move freely. The respiration patterns of the mice were videotaped. A trained operator performed the analysis frame-by-frame of movies off-line [80]. Measurement was made by counting the number of thoracic extensions of the animal within one minute. The test was conducted at 0, 5, 30, 60, 120, 180, 240, and 300 min after the injection of vehicle or 3-CMC.

#### 4.3.2. Body Temperature

To assess the effects of the substance on thermoregulation, we measured changes in the body’s core temperature. The core temperature was evaluated by a probe (1 mm diameter) that was gently inserted after lubrication into the rectum of the mouse (to about 2 cm) and left in position until temperature stabilization (about 10 s) [16]. The probe was connected to a Cole Parmer digital thermometer (model 8402). Measurement was conducted at 0, 15, 40, 70, 130, 190, 250, and 310 min after the treatment.

#### 4.3.3. Visual Responses

To evaluate the ability of the animal to capture visual information, two different tests were performed: the visual placing response, with mice freely moving, and the visual object response, when the animal is stationary [80]. Tests were performed at 0, 15, 40, 70, 130, 190, 250, and 310 min after the injection of vehicle or 3-CMC.

The visual placing test is performed using a tail suspension modified apparatus, which brings down the mouse towards the floor (constant speed of 10 cm/s). The downward movement of the mouse was videotaped by a camera placed at the base of the apparatus. A trained operator who was unaware of the drug treatments analyzed the videotapes. The frame-by-frame analysis allowed for the evaluation of the beginning of the mouse’s reaction when approaching the floor. As the mouse perceived the floor, it responded by extending the front paws, and an electronic ruler measured the perpendicular distance (mm) to the floor at which the mouse started the reaction.

The visual object test was performed to evaluate the mice’s ability to respond to the sight of an approaching object (from the front, for the frontal view, and from the side, for the lateral view). Usually, the response of mice is a head turn or retraction. For the frontal visual response, a white horizontal bar was moved frontally to the mouse head. The maneuver was repeated 3 times. For the lateral visual response, a small dentist’s mirror was moved into the mouse’s field of view in a horizontal arc. The procedure was conducted bilaterally and was repeated 3 times. The score assigned was 1 if there was a reflex or 0 if absent (overall score: 9).

#### 4.3.4. Acoustic Responses

The acoustic response test consists of the measurement of the mouse’s reflex to an acoustic stimulation produced behind the animal [79]. Four stimuli of different intensities and frequencies are produced: a snap of the finger, a sharp click (produced by a metal instrument), an acute sound (produced by an audiometer, frequency = 5.0–5.1 kHz and intensity = 96 dBA), and a severe sound (produced by an audiometer, frequency = 125–150 Hz and intensity = 86 dBA). Each stimulus was repeated three times, and a score of 1 was assigned in the presence of a response by the mouse (i.e., the turn or the shift of the head, the increase in breath rate, or by adopting a defence position). The total score was calculated by adding the scores obtained from each of the four tests (overall score: 12). The test was performed at 0, 15, 40, 70, 130, 190, 250, and 310 min after the injection of vehicle or 3-CMC.

#### 4.3.5. Tactile Responses

The tactile responses of mice treated with 3-CMC (0.1–30 mg/kg) were assessed through vibrissae, corneal, and pinnae reflexes [79] by using a gavage needle.

Vibrissae reflex was evaluated by touching the vibrissae (right and left) once on each side. A score of 1 was given if a response (head turn) was present, and a score of 0 was given if absent (overall score: 2). The corneal reflex was assessed by gently touching the cornea of the mouse. A value of 1 was assessed if the mouse responded by closing the eyelid, a value of 0 if there was no response. The test was repeated 3 times and was conducted bilaterally (overall score: 6). Pinna reflex was assessed by touching the pavilions (left and right), first the interior and then the external pavilions. The test was repeated twice per side, assigning a value of 1 if there was a reflex and 0 if absent (overall score: 4). Data are expressed as the sum of the three parameters (overall score: 12). The test was performed at 0, 15, 40, 70, 130, 190, 250 and 310 min after the injection of vehicle or 3-CMC.

#### 4.3.6. Motor Activity

The effect of 3-CMC on the spontaneous motor activity of mice was evaluated through the mobility time test [80], in which the animals were free to move in a square plastic cage (60 × 60 cm). A camera recorded the mouse activity, and videos were analysed offline by a trained operator, who measured (in seconds) the total time in motion of the animal over a period of 5 min by using a stopwatch. The test was performed at 0, 10, 35, 65, 125, 185, 245, and 305 min after treatment.

The accelerod test was used to assess alterations in stimulated motor activity induced by 3-CMC [79]. Mice were placed on the rotarod, an apparatus consisting of a rotating cylinder that automatically increases the speed of rotation in a constant manner (0–60 rotations/min in 5 min). Time spent on the rotarod during a 5 min period was measured by using a stopwatch. The test was performed at 0, 25, 50, 80, 140, 200, 260, and 320 min after the treatment.

The drag test was used to assess alteration in motor coordination induced by 3-CMC [79]. Each mouse was lifted by the tail, leaving the front paws on the table, and dragged backward at a constant speed (about 20 cm/s) for a fixed distance (100 cm). The number of steps performed by each paw was recorded by two different observers. The drag test was performed at 0, 25, 50, 80, 140, 200, 260, and 320 min post-injection.

#### 4.3.7. Prepulse Inhibition (PPI) Test

A different group of mice was tested for acoustic startle reactivity in a startle chamber (Ugo Basile apparatus, Milan, Italy) consisting of a sound-attenuated, lighted, and ventilated enclosure holding a transparent non-restrictive Perspex^®^ cage (90 × 45 × 50 mm). Acoustic stimuli were produced by a loudspeaker mounted laterally to the holder. Peak and amplitudes of the startle response were detected by a load cell. At the onset of the startling stimulus, 300-ms readings were recorded, and the wave amplitude evoked by the movement of the animal’s startle response was measured. The acoustic startle test session consisted of startle trials (pulse-alone) and prepulse trials (prepulse  +  pulse). The pulse-alone trials consisted of a 40-ms 120-dB pulse. Prepulse  +  pulse trials sequence consisted of a 20-ms acoustic prepulse, an 80-ms delay, followed by a 40-ms 120-dB startle pulse (100-ms onset-onset). Between the trials, there was an average of 15 s (range: from 9 to 21 s). Each startle session began with a 10 min acclimation period with a 65-dB broadband white noise, continuously present throughout the session. The test session contained 40 trials composed of pulse-alone and prepulse  +  pulse trials, with three different prepulses (68 dB, 75 dB, and 85 dB) presented in a pseudorandomized order [36]. Animals were placed in the startle chambers 5 min after treatment with vehicle or 3-CMC (1 and 10 mg/kg), and the entire PPI test lasted 20 min. The prepulse inhibition (% PPI) was expressed as the percentage decrease in the amplitude of the startle reactivity caused by the presentation of the prepulse.

### 4.4. Statistical Analysis

Data are represented as % of basal values (breath rate, visual placing response, mobility time, and rotarod), as Δ°C of basal values (body core temperature), and as arbitrary units (visual object, acoustic, and overall tactile responses). The amount of PPI was calculated as a percentage score for each prepulse  +  pulse trial type:

$$\%\;PPI\;=\;100\;-\;\left\{\left[\frac{startle\;response\;for\;prepulse\;+\;pulse\;trial}{startle\;response\;for\;pulse-alone\;trial}\right]\;\times \;100\right\}$$

Startle magnitude was calculated as the average response to all the pulse-alone trials. All data are shown as mean ± SD of 6 independent experimental replications. Two-way RM ANOVA followed by Bonferroni’s test for multiple comparison was employed for the statistical analysis of the effects of 3-CMC (0.1–30 mg/kg; i.p.) over time, while Two-way ANOVA followed by Bonferroni’s test for multiple comparison was used to compare the mean effects induced by each dose in female and male mice. One-way ANOVA followed by Bonferroni’s test was used for the statistical analysis of the effect of 3-CMC (1 and 10 mg/kg; i.p.) on Startle amplitude, and the Student’s *t*-test was used to evaluate sex differences in the effects of 3-CMC. On the other hand, Three-way ANOVA followed by Bonferroni’s test for multiple comparisons was used to evaluate the effect of 3-CMC on PPI in male and female mice at different prepulse intensities. All statistical analyses were performed with the Prism software 8.0 (GraphPad Prism, San Diego, CA, USA).

### 4.5. Case Series

In the current study, we described clinical data related to human 3-CMC-provoked intoxications over an 11-year period, with the aim of providing a translational slant, focusing on the Italian clinical scenario.

Regarding the study design, the presently employed protocol was approved, and clinical data were recorded/analysed, under the National Early Warning System (NEWS-D–Sistema Nazionale di Allerta Precoce-Department for Antidrug Policies-Presidency of the Italian Council of Ministers, Rome), actuated since 2008 in compliance with EU Decision 2005/387/JHA [81], in which the PCC-National Toxicology Information Centre (Istituti Clinici Scientifici Maugeri, IRCCS Pavia, Italy) is the National Coordinating Centre for clinic-toxicological aspects, strictly cooperating with international organisations of the EU EWS about news and alert on NPS. This interdisciplinary investigation was also reviewed and approved by the Ethics Committee of Istituti Clinici Scientifici Maugeri Hospital (1099 CE/2015 and 2615 CE/2022).

### 4.6. Patients, Biological Specimens, and Toxicological Examinations

This observational case series considers severely intoxicated subjects admitted to Italian Emergency Departments (EDs) and Intensive Care Units (ICUs) under the NEWS-D Project, from January 2014 to February 2025, for a clinically suspected abuse of a synthetic stimulant of the cathinone family, namely 3-CMC (3-chloromethcathinone), for which the PCC was contacted. On hospital admission, patients were informed about the project and signed the consent forms, prepared under the regulation established by the Department for Antidrug Policies-Presidency of the Italian Council of Ministers, Rome. Their biological samples (i.e., urine and blood) were collected and sent under refrigerated conditions to our laboratory for toxicological investigation; samples were stored at −80 °C until analyses. The standard analytical procedure included a non-targeted toxicological screening of urine or serum by GC-MS, followed by high-performance liquid chromatography-tandem mass spectrometry (LC-MS/MS). Also, the use of dedicated, updated electronic libraries was exploited (for details see following Section Analytical Determinations and Instrumentation) [82,83].

During the early consultation with ED emergency physicians, PCC medical toxicologists keep as much information as possible, recording data about the specific psychoactive substance/product brand name involved, dose, time, and route of intake, anamnestic data, and main outcomes. Next, a duplicate of the medical record, including full documentation of symptoms/management/treatment, was obtained. All clinical and laboratory data were anonymized for the scientific evaluation, and each individual case was associated with a code number.

#### Analytical Determinations and Instrumentation

Biological specimens from intoxicated patients were processed by high-performance analytical instruments, used for the toxicological quantitative confirmation analyses: (i) an Agilent GC-MS system (Agilent 7890A GC (Agilent Technologies, Santa Clara, CA, USA) coupled to a 5975 C MS detector (Agilent Technologies, Santa Clara, CA, USA), equipped with an Agilent Ultra 2 column (12 m, 0.2 mm diameter, 0.33 μm particle size; Agilent Technologies, Santa Clara, CA, USA) was used), operating in both SIM and SCAN modes, with the injector and detector temperatures at 260 and 300 °C, respectively (ii) a Shimadzu HPLC-UV/FL system (Shimadzu Italia S.r.l., Milan, Italy) and (iii) a Shimadzu LC-MS/MS 8050 (Shimadzu Italia S.r.l., Milan, Italy), operating in multiple reaction monitoring mode, with electrospray ionization source, also with the support of electronic libraries, including the software LC-MS/MS Shimadzu Forensic Toxicology DB (EN; LabSolution ver. 5.123) for the identification of untargeted compounds. LC-MS-MS detection was based on two product ions for each analyte obtained from the tuning of the relative certified standard in positive Electrospray Ionization (ESI+). The LC-MS-MS system consisted of a Shimadzu LC-MS/MS 8050 triple quadrupole operating in multiple reaction monitoring mode, with an electrospray ionization source. Chromatographic separation was performed on an Xbridge C18 column (3.5 µm, 2.1 × 100 mm; Waters SpA, Sesto San Giovanni, Italy) operating in gradient mode. The mobile phases were the following: (A) 5 mM ammonium formate in water, pH 3 with formic acid; (B) 0.1% formic acid in acetonitrile. The gradient started from 95% A, 5% B, got up to 20% A, 80% B in 10 min, and returned to the original conditions in 6 min. Flow rate was 0.2 mL/min. MS spectrometer operated in positive ionization and MRM mode with two transitions for each analyte. Using the current methods, it was not possible to distinguish isomers. The identification of the compound was attributed to the used reference standards [82,83].

### 4.7. In Silico ADMET Prediction

The in silico ADMET prediction of 2-CMC, 3-CMC, 4-CMC, 2-MMC, N-demethyl-3-CMC, and dihydro-3-CMC profiles was conducted through Simulations Plus ADMET Predictor^®^ Version 10.4 (×64) on a Windows 11 operating system (ADMET Predictor^®^, Simulations-Plus Inc; Lancaster, CA, USA). The program predicts the physicochemical, pharmacokinetic, and toxicity properties of compounds based on their molecular structures. It uses artificial neural network ensemble (ANNE) models, which were trained to ensemble with data sets that share the same “architecture” (i.e., same inputs and number of neurons) from well-defined drugs, using the 2D structure and the atomic descriptors for data selection [16]. For technical details of the methods used and for the criteria used to set the ADMET scores, see Supplementary Material (Tables S3–S6).

## 5. Conclusions

From our analysis, the acute administration of 3-CMC induced in mice relevant sensorial effects only at high doses. Some sex differences were observed in the physiological alterations (breath rate, core temperature) and motor activity. More in-depth studies are needed to clarify the reasons behind this, which may result from both pharmacodynamic and pharmacokinetic differences, and could be driven by sexual hormones [84]. Besides sex differences, it is worth noting that the locomotory stimulation and the increase in the breath rate observed in mice are in line with the psychomotor agitation and tachypnoea reported for some of the intoxicated patients. Moreover, the observed PPI impairment is indicative of the alteration of sensorimotor gating, which is related to the psychotic events and hallucinations described. In this regard, sex resulted in having an impact on the effect of 3-CMC on PPI, which possibly suggests that female users might be more sensitive to psychotic effects after the assumption of 3-CMC.

Despite the similar absorption and transport properties, it emerged that a minimal structural difference can alter some important ADMET properties, as the hepatotoxicity potential and interaction with CYPs. At present, it is not known whether 3-CMC metabolites are substrates/inhibitors at DAT, NET, and SERT; however, the in silico investigation indicates that they can cross the BBB and present a toxic potential themselves, possibly contributing to the health risks associated with the consumption of 3-CMC, thus opening new objectives for future studies.

## 6. Limitations

At present, most of the 3-CMC intoxication reports involve male subjects, which prevents evaluating any sex-tailored differences in the effects experienced with the compound. Moreover, in the in vivo preclinical study, we did not perform any maneuver to synchronize the menstrual cycle of female mice, with the aim of better representing the overall female population. Another limitation is that in the near majority of reported cases, different substances were identified, which complicates the correlation between the effects of 3-CMC alone in humans and mice. Finally, we are aware that it would be interesting to test the effects of a 30 mg/kg dose on PPI; however, given the availability of 3-CMC and the reduction principle for animal research (3Rs principles), we did not perform it. Based on the results obtained with the 10 mg/kg dose, we hypothesize that a similar effect would be observed with increasing dose, as previously observed [85]. However, future studies will eventually confirm that.

## Figures and Tables

**Table 2:** CMC human poisonings (Pavia Poison Centre-National Toxicology Information Centre data) over an 11-year interval (2014–2025). All events were recorded in the two-year period 2022/2023. No intoxication case was documented between 2014 and 2021, nor in 2024–2025. All cases were intentional non-fatal intoxications.

*Case n./Sex, Age*	*EDs Admission*	*Reported/* *Suspected Substance(s)*	*Reported Route of Intake*	*On-Site Screening Positivity*	*Signs/Symptoms*	*Patient Management*	Blood/Urine: Confirmatory Analyses (*) *(§, ^, °°, ^●^, $)*	*Anamnestic Data,* *Annotations,* *Hospital Stay/Outcomes*	*NEWS Alert*
#**1** 23, M	*January 2022*	Mephedrone + BZO (Tavor)	oral	−	CNS alterations (psychomotor agitation, mental confusion, aggressiveness, psychosis–self-injuring, i.e., attempted suicide)	Symptomatic treatment, Sedation-Benzodiazepines (midazolam), psycholeptic/antipsychotic drug (Clotiapine), hydration	CMC (§)	Diagnosed for borderline personality disorder; long-lasting abuser (self-referred abuse of COC, MTD, Mephedrone, ethanol) Suspected sexual context (ChemSex) Taken to ED by ambulance staff <24 h, transferred to psychiatric ward;	*SNAP 13/22 15 April 2022*
#**2** 43, M	*March 2023*	Popper + 3-MMC	nasal	−	CNS alterations (psychomotor agitation), thoracic pain, tachycardia, respiratory distress, tachypnoea, desaturation (88% in ambient air); MethHb (14%)	Symptomatic treatment, Methylene blue (60 mg iv)	3-CMC + nitrite/nitrate ions (**) (^)	self-referred thoracic trauma during sexual activity, suspected sexual context (ChemSex) <24 h, discharged home	*SNAP. 30/23 27 July 2023*
#**3** 43, M	*April 2023*	Mephedrone + ketamine	nasal + im + iv	COC	CNS alterations (psychomotor agitation, mental confusion, hallucinations), respiratory alkalosis, cardiac arrhythmias (tachycardia and extrasystoles), mydriasis	Symptomatic treatment, Benzodiazepines (midazolam), magnesium sulphate, hydration	3-CMC + 2-FDCK (°°)	Suspected sexual context (ChemSex) <24 h, discharged home	*SNAP 15/24 18 March 2024*
#**4** 25, M	*April 2023*	GHB + Ethanol	oral	AMP/ MAMP	CNS depression/coma, bradycardia, miosis, vomiting	Symptomatic treatment, suctioning, gastric decontamination, hydration	3-CMC + Mephedrone isomer + GHB (^●^)	GHB assumption in a nightclub, suspected sexual context (ChemSex) Taken to ED by ambulance staff <24 h, discharged home	*SNAP. 30/23 27 July 2023*
**#5** 37, M	*May 2023*	α-PVP	smoke	BZO	CNS alterations (psychomotor agitation, mental confusion)	Symptomatic treatment, Benzodiazepines (midazolam), Neuroleptic drug (Promazine), hydration	MDPHP + CMC ($)	Occasional NPS user, suspected sexual context (ChemSex) Taken to ED by police agency and ambulance staff About 30 h, discharged home	*SNAP. 30/23 27 July 2023*

−: information missing; oral: *ingestion*; nasal: “sniffing” or “snorted”; im: *intramuscular injection*; iv: *intravenous injection*; BZO: *Benzodiazepines*; CMC: *Chloromethcathinone*; COC: *Cocaine*; MTD: *Methadone*; 3-MMC: *3-Methylmethcathinone*; MethHb: methemoglobinemia; 3-CMC: 3-Chloromethcathinone; 2-FDCK: *2-Fluorodeschloroketamine*; GHB: *gamma hydroxybutyrate*; AMP/MAMP: *Amphetamines/Methamphetamine*; α-PVP: *alpha-pyrrolidinovalerophenone*; MDPHP: *3,4-methylenedioxy-alpha-pyrrolidinohexanophenone*; SNAP: *Sistema Nazionale Allerta Precoce–Italia*. (*) GC-MS and LC-MS methods (based on standard availability). (**) Nitrites/Nitrates determination performed by the Department of Diagnostic and Public Health, Section of Forensic Medicine, University of Verona. (§) tested negative for the following substances: butylone, mephedrone (4-MMC), dimethylcathinone, dimethylmethcathinone, buphedrone, ethcathinone, 4-fluoromethcathinone, pentedrone, methedrone, mexedrone, etylone, pentylone, 1-naphyrone, eutylone, ephylon, methylcathinone, N-etylpentedrone, MDPEP, 3,4-MDPHP, α-PVP, MDPV, betaK-2CB, PMA, PMMA, 4-fluoroamphetamine, 4-MTA, ketamine, levamisole, scopolamine, atropine, methylphenidate, ethylphenidate, 4-fluoromethylphenidate, N,N-Dimethyltryptamine (DMT), (aminopropyl)benzofurans (APB), methoxetamine, methoxyphenidine, diphenidine, 2CI, 2CT7, 2CB, 2CT2, 2CE, DOB, 25B-NBOME, 25C-NBOME, 25I-NBOME, 25T7-NBOME, 25H-NBOME, 25D-NBOME, 25E-NBOME, Phencyclidine (PCP), fluoroketamine, OH-PCP, OH-PCE, MeO-PCE, MeO-PCP, OXO-PCE, 5-EAPB, 5/6-MAPB, 4-OH-DET, 5-MeO-DALT, 5-Meo-DMT, 5-MeOMiPT. (^) tested negative for the following substances: butylone, mephedrone (4-MMC), dimethylcathinone, dimethylmethcathinone, buphedrone, ethcathinone, 4-fluoromethcathinone, pentedrone, methedrone, mexedrone, etylone, pentylone, 1-naphyrone, eutylone, ephylon, clephedrone, methylcathinone, N-etylpentedrone, MDPEP, 3,4-MDPHP, α-PVP, MDPV, betaK-2CB, PMA, PMMA, 4-fluoroamphetamine, 4-MTA, ketamine, levamisole, scopolamine, atropine, methylphenidate, ethylphenidate, 4-fluoromethylphenidate, N,N-Dimethyltryptamine (DMT), (aminopropyl)benzofurans (APB), methoxetamine, methoxyphenidine, diphenidine, 2CI, 2CT7, 2CB, 2CT2, 2CE, DOB, 25B-NBOME, 25C-NBOME, 25I-NBOME, 25T7-NBOME, 25H-NBOME, 25D-NBOME, 25E-NBOME, Phencyclidine (PCP), fluoroketamine, OH-PCP, OH-PCE, MeO-PCE, MeO-PCP, OXO-PCE, 5-EAPB, 5/6-MAPB, 4-OH-DET, 5-MeO-DALT, 5-Meo-DMT, 5-MeOMiPT, 5-MMPA. (°°) tested negative for the following substances: butylone, mephedrone (4-MMC), dimethylcathinone, dimethylmethcathinone, buphedrone, ethcathinone, 4-fluoromethcathinone, pentedrone, methedrone, mexedrone, etylone, pentylone, 1-naphyrone, eutylone, ephylon, clephedrone, methylcathinone, N-etylpentedrone, MDPEP, 3,4-MDPHP, α-PVP, MDPV, betaK-2CB, PMA, PMMA, 4-fluoroamphetamine, 4-MTA, ketamine, levamisole, scopolamine, atropine, methylphenidate, ethylphenidate, 4-fluoromethylphenidate, N,N-Dimethyltryptamine (DMT), (aminopropyl)benzofurans (APB), methoxetamine, methoxyphenidine, diphenidine, 2CI, 2CT7, 2CB, 2CT2, 2CE, DOB, 25B-NBOME, 25C-NBOME, 25I-NBOME, 25T7-NBOME, 25H-NBOME, 25D-NBOME, 25E-NBOME, Phencyclidine (PCP), OH-PCP, OH-PCE, MeO-PCE, MeO-PCP, OXO-PCE, 5-EAPB, 5/6-MAPB, 4-OH-DET, 5-MeO-DALT, 5-Meo-DMT, 5-MeOMiPT, 5-MMPA. (^●^) tested negative for the following substances: butylone, mephedrone (4-MMC), dimethylcathinone, dimethylmethcathinone, buphedrone, ethcathinone, 4-fluoromethcathinone, pentedrone, methedrone, mexedrone, etylone, pentylone, 1-naphyrone, eutylone, ephylon, clephedrone, methylcathinone, N-etylpentedrone, MDPEP, 3,4-MDPHP, α-PVP, MDPV, betaK-2CB, PMA, PMMA, 4-fluoroamphetamine, 4-MTA, ketamine, levamisole, scopolamine, atropine, methylphenidate, ethylphenidate, 4-fluoromethylphenidate, N,N-Dimethyltryptamine (DMT), (aminopropyl)benzofurans (APB), methoxetamine, methoxyphenidine, diphenidine, 2CI, 2CT7, 2CB, 2CT2, 2CE, DOB, 25B-NBOME, 25C-NBOME, 25I-NBOME, 25T7-NBOME, 25H-NBOME, 25D-NBOME, 25E-NBOME, Phencyclidine (PCP), fluoroketamine, OH-PCP, OH-PCE, MeO-PCE, MeO-PCP, OXO-PCE, 5-EAPB, 5/6-MAPB, 4-OH-DET, 5-MeO-DALT, 5-Meo-DMT, 5-MeOMiPT, 5-MMPA. ($) tested negative for the following substances: butylone, mephedrone (4-MMC), dimethylcathinone, dimethylmethcathinone, buphedrone, ethcathinone, 4-fluoromethcathinone, pentedrone, methedrone, mexedrone, etylone, pentylone, 1-naphyrone, eutylone, ephylon, methylcathinone, N-etylpentedrone, MDPEP, α-PVP, MDPV, betaK-2CB, PMA, PMMA, 4-fluoroamphetamine, 4-MTA, ketamine, levamisole, scopolamine, atropine, methylphenidate, ethylphenidate, 4-fluoromethylphenidate, N,N-Dimethyltryptamine (DMT), (aminopropyl)benzofurans (APB), methoxetamine, methoxyphenidine, diphenidine, 2CI, 2CT7, 2CB, 2CT2, 2CE, DOB, 25B-NBOME, 25C-NBOME, 25I-NBOME, 25T7-NBOME, 25H-NBOME, 25D-NBOME, 25E-NBOME, Phencyclidine (PCP), fluoroketamine, OH-PCP, OH-PCE, MeO-PCE, MeO-PCP, OXO-PCE, 5-EAPB, 5/6-MAPB, 4-OH-DET, 5-MeO-DALT, 5-Meo-DMT, 5-MeOMiPT, 5-MMPA.

**Table 3:** Prediction of CYPs involved in metabolism of tested compounds and percentage of accuracy (%) by ADMET predictor^®^.

Drug	1A2 Substr	1A2 Inh	2A6 Substr	2A6 Inh	2C8 Substr	2C8 Inh	2C9 Substr	2C9 Inh	2C19 Substr	2C19 Inh	2D6 Substr	2D6 Inh	2E1 Substr	2E1 Inh	3A4 Substr	3A4 Inh
3-CMC	**Yes** **(66%)**	**Yes** **(55%)**	**Yes** **(42%)**	No (99%)	**Yes** **(59%)**	No (97%)	No (94%)	No (97%)	**Yes** **(81%)**	No (96%)	**Yes** **(71%)**	No (74%)	**Yes** **(97%)**	No (99%)	No (37%)	No (96%)
2-CMC	**Yes** **(69%)**	**Yes** **(46%)**	**Yes** **(42%)**	No (99%)	**Yes** **(59%)**	No (97%)	No (94%)	No (97%)	**Yes** **(81%)**	No (96%)	**Yes** **(58%)**	No (79%)	**Yes** **(97%)**	No (99%)	No (66%)	No (96%)
4-CMC	**Yes** **(69%)**	**Yes** **(59%)**	**Yes** **(42%)**	No (98%)	**Yes** **(58%)**	No (97%)	No (94%)	No (97%)	**Yes** **(81%)**	No (96%)	**Yes** **(71%)**	No (81%)	**Yes** **(97%)**	No (99%)	No (49%)	No (96%)
2-MMC	**Yes** **(91%)**	No (83%)	**Yes** **(40%)**	No (99%)	No (57%)	No (97%)	No (96%)	No (97%)	**Yes** **(66%)**	No (96%)	**Yes** **(87%)**	No (84%)	**Yes** **(64%)**	No (99%)	No (68%)	No (96%)
N-demethyl-3-CMC	**Yes** **(63%)**	**Yes** **(47%)**	**Yes** **(40%)**	No (87%)	No (91%)	No (77%)	No (99%)	No (97%)	**Yes** **(60%)**	No (76%)	No (63%)	No (76%)	**Yes** **(92%)**	No (97%)	No (68%)	No (96%)
dihydro-3-CMC	No (50%)	No (96%)	No (98%)	No (99%)	**Yes** **(56%)**	No (97%)	No (99%)	No (97%)	**Yes** **(81%)**	No (96%)	**Yes** **(87%)**	No (78%)	**Yes** **(92%)**	No (99%)	No (61%)	No (96%)

Positive results are in bold format.

**Table 4:** Predicted permeability and transporting properties and their percentage of accuracy (%) by ADMET predictor^®^.

Drug	S + Peff ^a^	S + MDCK ^b^	Vd ^c^	BBB Penetration ^d^	Pgp Sub ^e^	Pgp Inh ^f^	OCT1 Sub ^g^	OCT1 Inh ^h^	OCT2 Sub ^i^	OCT2 Inh ^j^
3-CMC	4.542	1756.472	6.003	High (99%)	No (50%)	No (93%)	No (89%)	No (64%)	No (56%)	**Yes** (52%)
2-CMC	4.607	1742.266	5.693	High (99%)	No (47%)	No (93%)	No (89%)	No (76%)	No (52%)	**Yes** (49%)
4-CMC	4.534	1640.429	6.5	High (99%)	No (50%)	No (93%)	No (89%)	No (64%)	No (50%)	**Yes** (69%)
2-MMC	3.629	1492.15	5.028	High (99%)	**Yes ** (58%)	No (93%)	No (89%)	No (61%)	**Yes** (75%)	**Yes** (39%)
N-demethyl-3-CMC	3.651	1077.269	4.35	High (99%)	No (47%)	No (93%)	No (89%)	No (76%)	No (58%)	**Yes** (46%)
dihydro-3-CMC	2.938	1169.019	4.321	High (96%)	**Yes ** (77%)	No (93%)	**Yes** (56%)	**Yes** (53%)	**Yes ** (75%)	**Yes** (69%)

^a^ Jejunal permeability [cm/s × 10^4^]; ^b^ Madin–Darby canine kidney [cm/s × 10^7^]; ^c^ volume of distribution [L/Kg]; ^d^ blood–brain barrier; ^e^ P-glycoprotein substrate; ^f^ P-glycoprotein inhibitor; ^g^ organic cation transporting 1 substrate; ^h^ organic cation transporting 1 inhibitor; ^i^ organic cation transporting 2 substrate; ^j^ organic cation transporting 2 inhibitor. Positive results are in bold format.

**Table 5:** Predicted effects and their percentage of accuracy (%) by ADMET Predictor^®^ on hepatic enzyme levels in human serum and on hERG potassium channel.

Drug	Ser_AlkPhos ^a^	Ser_GGT ^b^	Ser_LDH ^c^	Ser_AST ^d^	Ser_ALT ^e^	HEP hCLint ^f^	hERG Filter ^g^	hERG pIC50 ^h^
3-CMC	Normal (83%)	Normal (75%)	**Elevated** **(68%)**	**Elevated** **(44%)**	**Elevated (53%)**	13.24	**Yes** (86%)	5.132
2-CMC	Normal (68%)	Normal (75%)	**Elevated** **(68%)**	**Elevated** **(60%)**	**Elevated (69%)**	13.413	**Yes** (82%)	5.113
4-CMC	Normal (92%)	Normal (75%)	**Elevated** **(68%)**	Normal (45%)	Normal (53%)	15.31	**Yes** (86%)	5.12
2-MMC	Normal (92%)	**Elevated** **(46%)**	**Elevated** **(55%)**	Normal (78%)	Normal (81%)	7.696	**Yes** (57%)	4.923
N-demethyl-3-CMC	Normal (92%)	Normal (82%)	**Elevated** **(68%)**	**Elevated** **(56%)**	**Elevated (80%)**	7.315	**Yes** (50%)	5.063
dihydro-3-CMC	Normal (61%)	Normal (77%)	**Elevated** **(51%)**	**Elevated** **(50%)**	Normal (69%)	5.805	**Yes ** (54%)	5.256

^a^ serum alkaline phosphatase; ^b^ serum Gamma-glutamyl transferase; ^c^ serum lactate dehydrogenase; ^d^ serum aspartic acid transaminase; ^e^ serum alanine transaminase; ^f^ intrinsic clearance in μL/min/million cells for metabolism in human hepatocytes (unbound form); ^g^ potassium channel block; ^h^ affinity to the hERG potassium channel in human expressed as pIC_50 in mol/L. Positive results are in bold format.

## Data Availability

The original contributions presented in this study are included in the article/Supplementary Material. Further inquiries can be directed to the corresponding author.

## Supplementary Materials

The following supporting information can be downloaded at: https://www.mdpi.com/article/10.3390/ijms262311600/s1.

## Abbreviations

The following abbreviations are used in this manuscript:

5-HT	Serotonin
ADMET	Absorption, Distribution, Metabolism, Elimination, Toxicity
CMCs	Chloro-methcathinones
CNS	Central Nervous System
DA	Dopamine
DAT	Dopamine transporter
MMCs	Methyl-methcathinones
NE	Norepinephrine
NET	Norepinephrine transporter
NEWS-D	Italian National Drug Early Warning System
NPS	Novel Psychoactive Substances
SCs	Synthetic cathinones
SERT	Serotonin transporter
