# Supplementary material for: 3-CMC: Acute Effects in Male and Female Mice, Human Intoxication Case Series (Italy, 2014–2025), and Prediction of ADMET Properties
Source: Int J Mol Sci. 2025 Nov 29;26(23):11600. doi: 10.3390/ijms262311600 (PMC12692235; doi:10.3390/ijms262311600)
Supplement: Supplementary file 1 [file ijms-26-11600-s001.zip › ijms-3955210-supplementary.pdf]

Supplementary material

3-CMC: acute effects in male and female mice, human intoxication case series (Italy, 2014-2025), and prediction of ADMET properties

Marta Bassi [1], Elisa Roda [2], Giorgia Corli [1], Sabrine Bilel [1], Fabrizio De Luca [3], Tatiana Bernardi [4], Adolfo Gregori [5], Fabiana Di Rosa [6], Davide Lonati [2], Carlo Alessandro Locatelli [2] and Matteo Marti [1,7,*]

[*]***Corresponding Author***: Department of Translational Medicine, Section of Legal Medicine, University of Ferrara

via Fossato di Mortara 70, 44121 Ferrara Italy

phone +39 0532 455781, fax +39 0532 455777

email: mto@unife.it

Supplementary Results

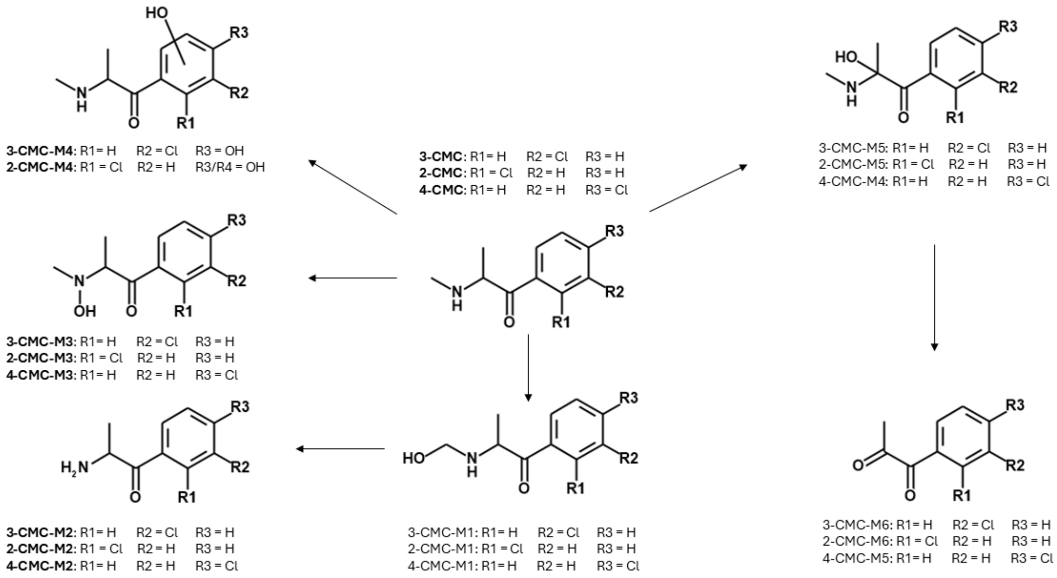

Figure S1. Predicted metabolism of 3-CMC, 2-CMC and 4-CMC by ADMET Predictor®. The main predicted metabolites are shown in bold font.

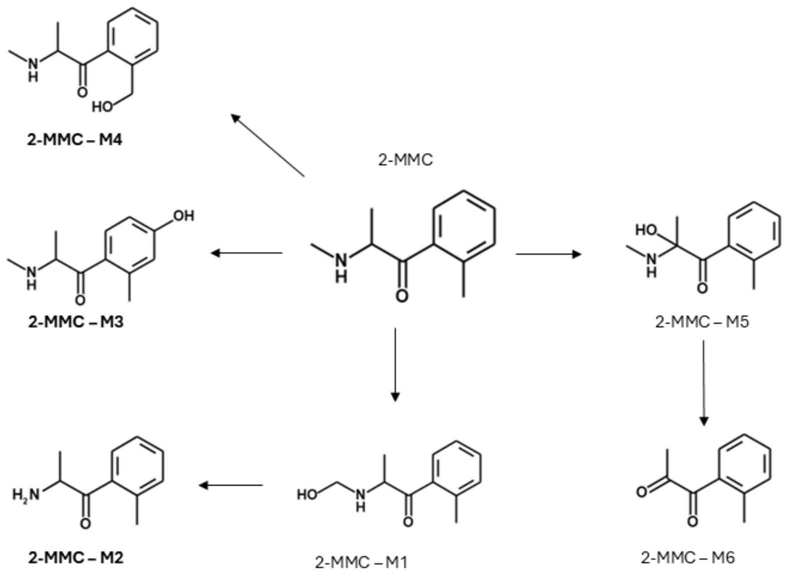

Figure S2. Predicted metabolism of 2-MMC by ADMET Predictor®. The main predicted metabolites are shown in bold font.

Table S1. *Results of statistical analysis with Two-way RM ANOVA followed by Bonferroni's test for multiple comparisons of the behavioural tests conducted on male and female CD-1 mice treated with vehicle or 3-CMC (0.1-30 mg/kg; i.p.).*

	**BREATH RATE**					
	**Female mice**			**Male mice**		
	**F (DFn, DFd)**	**p value**	**Geisser-Greenhouse's epsilon**	**F (DFn, DFd)**	**P value**	**Geisser-Greenhouse's epsilon**
Subject	F(30, 210)=12.86	p<0.0001		F(30, 210)=21.26	p<0.0001	
Treatment	F(5, 30)=4.483	P=0.0036		F(5, 30)=6.863	p=0.0002	
Time	F(2.707, 81.20)=32.14	p<0.0001	0.3867	F(3.050, 91.51)=101.2	p<0.0001	0.4357
Interaction	F(35, 210)=3.625	p<0.0001		F(35, 210)=8.749	p<0.0001	
	**CORE TEMPERATURE**					
	**Female mice**			**Male mice**		
	**F (DFn, DFd)**	**p value**	**Geisser-Greenhouse's epsilon**	**F (DFn, DFd)**	**P value**	**Geisser-Greenhouse's epsilon**
Subject	F(30, 180)=29.35	p<0.0001		F(30, 180)=15.89	p<0.0001	
Treatment	F(5, 30)=10.82	p<0.0001		F(5, 30)=15.26	p<0.0001	
Time	F(3.571, 107.1)=35.92	p<0.0001	0.5951	F(3.201, 96.03)=42.71	p<0.0001	0.5335
Interaction	F(30, 180)=32.63	p<0.0001		F(30, 180)=15.89	p<0.0001	
	**VISUAL PLACING RESPONSE**					
	**Female mice**			**Male mice**		
	**F (DFn, DFd)**	**p value**	**Geisser-Greenhouse's epsilon**	**F (DFn, DFd)**	**P value**	**Geisser-Greenhouse's epsilon**
Subject	F(30, 210)=8.999	p<0.0001		F(30, 210)=18.35	p<0.0001	
Treatment	F(5, 30)=44.85	p<0.0001		F(5, 30)=58.65	p<0.0001	
Time	F(3.172, 95.16)=38.75	p<0.0001	0.4531	F(2.015, 60.45)=62.83	p<0.0001	0.2879
Interaction	F(35, 210)=50.78	p<0.0001		F(35, 210)=2482	p<0.0001	
	**VISUAL OBJECT RESPONSE**					
	**Female mice**			**Male mice**		
	**F (DFn, DFd)**	**p value**	**Geisser-Greenhouse's epsilon**	**F (DFn, DFd)**	**P value**	**Geisser-Greenhouse's epsilon**
Subject	F(30, 210)=3.677	p<0.0001		F(30, 210)=5.005	p<0.0001	
Treatment	F(5, 30)=11.51	p<0.0001		F(5, 30)=2.172	p=0.0839	
Time	F(2.536, 76.07)=15.51	p<0.0001	0.3622	F(2.964, 88.92)=7.979	p<0.0001	0.4234
Interaction	F(35, 210)=5.685	p<0.0001		F(35, 210)=2.933	p<0.0001	
	**ACOUSTIC RESPONSE**					
	**Female mice**			**Male mice**		
	**F (DFn, DFd)**	**p value**	**Geisser-Greenhouse's epsilon**	**F (DFn, DFd)**	**P value**	**Geisser-Greenhouse's epsilon**
Subject	-	-	-	F(30, 210)=3.888	p<0.0001	
Treatment	-	-	-	F(5, 30)=4.893	p=0.0022	
Time	-	-	-	F(2.016, 60.47)=2.788	p=0.0690	0.2879

Interaction	-	-	-	F(35, 210)=1.127	p=0.2975	
	**OVERALL TACTILE RESPONSE**					
	**Female mice**			**Male mice**		
	**F (DFn, DFd)**	**p value**	**Geisser-Greenhouse's epsilon**	**F (DFn, DFd)**	**P value**	**Geisser-Greenhouse's epsilon**
Subject	F(30, 210)=2.884	p<0.0001		F(30, 210)=2.063	P=0.0017	
Treatment	F(5, 30)=8.115	p<0.0001		F(5, 30)=40.30	p<0.0001	
Time	F(1.798, 53.94)=6.947	p=0.0028	0.2569	F(1.227, 38.80)=49.60	p<0.0001	0.1752
Interaction	F(35, 210)=6.947	p<0.0001		F(35, 210)=49.60	p<0.0001	
	**MOBILITY TIME**					
	**Female mice**			**Male mice**		
	**F (DFn, DFd)**	**p value**	**Geisser-Greenhouse's epsilon**	**F (DFn, DFd)**	**P value**	**Geisser-Greenhouse's epsilon**
Subject	F(30, 210)=12.01	p<0.0001		F(30, 210)=8.513	p<0.0001	
Treatment	F(5, 30)=13.93	p<0.0001		F(5, 30)=5.459	p=0.0011	
Time	F(3.342, 100.3)=147.7	p<0.0001	0.4774	F(4.458, 133.7)=398.7	p<0.0001	0.6368
Interaction	F(35, 210)=20.75	p<0.0001		F(35, 210)=9.312	p<0.0001	
	**ROTAROD TEST**					
	**Female mice**			**Male mice**		
	**F (DFn, DFd)**	**p value**	**Geisser-Greenhouse's epsilon**	**F (DFn, DFd)**	**P value**	**Geisser-Greenhouse's epsilon**
Subject	F(30, 210)=14.91	p<0.0001		F(30, 210)=18.26	p<0.0001	
Treatment	F(5, 30)=4.946	p=0.0020		F(5, 30)=5.924	p=0.0006	
Time	F(2.235, 67.06)=12.10	p<0.0001	0.3193	F(2.688, 80.64)=15.45	p<0.0001	0.384
Interaction	F(35, 210)=17.71	p<0.0001		F(35, 210)=24.68	p<0.0001	
	**DRAG TEST**					
	**Female mice**			**Male mice**		
	**F (DFn, DFd)**	**p value**	**Geisser-Greenhouse's epsilon**	**F (DFn, DFd)**	**P value**	**Geisser-Greenhouse's epsilon**
Subject	F(30, 210)=13.94	p<0.0001		F(30, 210)=18.42	p<0.0001	
Treatment	F(5, 30)=0.6150	p=0.6892		F(5, 30)=0.8391	p=0.5326	
Time	F(3.691, 110.7)=6.015	p=0.0003	0.5273	F(2.864, 85.93)=1.691	p=0.1771	0.4092
Interaction	F(35, 210)=5.406	p<0.0001		F(35, 210)=3.331	p<0.0001	

- : non-significant effect

Material and methods

1. Behavioural study

The effects induced in mice by 3-CMC (0.1-30 mg/kg, i.p.) on behavioral responses were investigated through a battery of tests (Safety Pharmacology) routinely employed in our laboratory for the preclinical characterization of new molecules [76,77]. With the aim to reduce the number of mice employed, the sensorimotor response tests were conducted consecutively according to the following time scheme: breath rate, mobility time, visual (placing and object) responses, acoustic responses, overall tactile responses, body core temperature, time on rod, and number of steps. The test battery was conducted at 0 (prior to administration), 5, 30, 60, 120, 180, 240, and 300 minutes after the treatment. For more detailed information about time points, see **Table S3**.

Experiments were carried out in a laboratory with controlled temperature (20-22°C), humidity (45-55%), light (150 lux), and background noise (40 ± 4 dB). The behavioural tests were conducted by blinded and trained operators working in pairs and videotaped by a camera (B/W USB Camera day&night with varifocal lens; Ugo Basile, Italy) placed at the top or on one side of the box and analysed offline by a different trained operator.

Table S2. *Description of the timetable of each test of the Safety Pharmacology battery of tests.*

	**TIME POINTS**							
	**0 (control, before the treatment)**	**5 min post inj**	**30 min post inj**	**60 min post inj**	**120 min post inj**	**180 min post inj**	**240 min post inj**	**300 min post inj**
Observation and breath rate measurement	0’ - 5’	0’ - 5’	30’ - 35’	60’ - 65’	120’ - 125’	180’ - 185’	240’ - 245’	300’ - 305’
Mobility time	5’ - 10’	5’ - 10’	35’ - 40’	65’ - 70’	125’ - 130’	185’ - 190’	245’ - 250’	305’ - 310’
Visual, acoustic, tactile test	10’ - 15’	10’ - 15’	40’ - 45’	70’ - 75’	130’ - 135’	190’ - 195’	250’ - 255’	310’ - 315’
Body core temperature measurement	15’ - 16’	15’ - 16’	45’ - 46’	75’ - 76’	135’ - 136’	195’ - 196’	255’ - 256’	315’ - 316’
Rotarod test	16’ - 21’	16’ - 21’	46’ - 51’	76‘ - 81’	136’ - 141’	196’ - 201’	256’ - 261’	316’ - 321’
Drag test	21’ - 26’	21’ - 26’	51’ - 56’	81’ - 86’	141’ - 146’	201’ - 206’	261’ - 266’	321’ - 326’
Total	26’	26’	26’	26’	26’	26’	26’	26’

2. In silico ADMET prediction

The *in silico* characterization of the ADMET profile of 3-CMC, 2-CMC, 4-CMC, 2-MMC, N-demethyl-3-CMC, and dihydro-3-CMC was conducted through Simulations Plus ADMET Predictor® Version 10.4 (×64) on a Windows 11 operating system. The program allows the prediction of ADMET properties based on the molecular structures of compounds. It uses artificial neural network ensemble (ANNE) models which were trained to ensemble with data sets that share the same "architecture" (i.e., same inputs and number of neurons) from well-defined drugs, using the 2D structure and the atomic descriptors for data selection. ADMET Predictor® models have been shown to have a similar or better accuracy when compared to other available software.

2.1. Evaluation of risks

As an initial screening step, all compounds were screened based on some of their calculated risk scores. The program provides a score, labelled “ADMET_Risk” that is a general score ranging from 0 to 24 which indicates the number of potential ADMET issues that a compound may face. A threshold of concern for each risk is suggested by the program and is calculated by defining the threshold below which 90% of drugs in the World Drug Index (WDI) score. In

addition, the compounds were assessed for their conformance to Lipinski's Rule of Five (RO5). According to RO5, a drug can be orally active only if it meets at least three of the following criteria: molecular weight (MW) <500 Daltons, hydrogen bond acceptors (HBA) ≤10, hydrogen bond donors (HBD) ≤5, logP ≤5 (13).

2.1.1. Absorption risk

The absorption risk model (Absn_Risk) includes eight rules based on descriptors and predicted properties licensed as part of the PhysChem model group, each of which contributes one vote to the score. The rules are illustrated in **Table S3**.

Table S3. *The eight rules based on descriptors and predicted properties used to assess absorption risk.*

Parameter	Rule of absorption risk
**Size**	MWt > [450,500] or N_Atoms > [30,35] or MolVol > [470,520] or N_Bonds > [35,40]
**RotB**	N_FrRotB > [8,10] (too flexible)
**HBD**	HBDH > [3,5] and HBDch > [1.5,2.0] (too many good H-bond donors)
**HBA**	HBA > [7,10] and HBAch < [-6.0,-5.0] (too many good H-bond acceptors)
**Charge**	NPA_ABSQ > [19,21] or T_PSA > [120 Å2,140 Å2] (excessive charge)
**Kow**	S+logP > [4.5,5.0] or S+logD > [3.5,4.0] OR MlogP > [3.5,4.0] (high logPoctanol-water)
**Peff**	S+Peff < [0.40,0.60] (low permeability)
**Sw**	S+Sw < [0.005,0.010] (low solubility)

RotB. *Rotational bonds;* ***N_FrRotB.*** *number of freely rotatable bonds;* ***HBD*** *hydrogen bond donors;* ***HBDH****. hydrogen bond donors hydrogens;* ***HBDch*** *hydrogen bond charge* ***HBA.*** *hydrogen bond acceptors;* ***NPA_ABSQ*** *Sum of absolute values of estimated NPA partial;* ***T_PSA*** *Topological polar surface area;* ***Kow.*** *n-Octanol/Water Partition Coefficient;* ***S+logP****. Octanol-water partition coefficient Simulations Plus;* ***S+logD,*** *Octanol-water distribution coefficient Simulations Plus [cm/s×10^4];* ***MlogP,*** *Moriguchi octanol-water partition coefficient.* ***Peff****, human jejunal permeability;* ***S+Peff.*** *human effective jejunal permeability Simulations Plus;* ***Sw,*** *Water solubility;* ***S+Sw,*** *Native water solubility; Simulations Plus*

2.1.2. Cytochrome risk

The cytochrome risk model is comprised of six rules, each with a weight of one illustrated in **Table S4.**

Table S4. *The six rules based on descriptors and predicted properties used to assess cytochrome risk.*

Parameter	Rule of cytochrome risk
**1A2**	CYP1A2_CLint > [20,40]
**2C9**	CYP2C9_CLint > [10,20]
**2C19**	CYP2C19_CLint > [20,40]
**2D6**	CYP2D6_CLint > [10,20]
**3A4**	CYP3A4_CLint > [20,50] and CYP3A4_HLM_CLint > [30,75]
**CLEARANCE**	CYP_HLM_CLint > [90,150] or HEP_hCLint > [60,90]

CYP1A2_CLint*, intrinsic clearance constant for CYP 1A2 mediated metabolism [μl/min/mg];* ***CYP2C9_CLint****. intrinsic clearance constant for CYP 2C9 mediated metabolism [μl/min/mg];* ***CYP_2C19_CLint****. intrinsic clearance constant for CYP 2C19 mediated metabolism [μl/min/mg];* ***CYP2D6_CLint****.intrinsic clearance constant for CYP 2D6 mediated metabolism [μl/min/m];* ***CYP3A4_CLint****. intrinsic clearance constant for CYP 3A4 mediated metabolism[μl/min/mg];* ***CYP_HLM_CLint:*** *overall in vitro (unbound) intrinsic clearance in Human Liver Microsomes [μ L/min/mg HLM protein];* ***HEP_hCLint:*** *overall in vitro (unbound) intrinsic clearance in human hepatocytes [μ L/min/10^6 cells].*

2.1.3. Toxicity risk

The toxicity risk model consists of five rules (**Table S5**), including one based on mutation risk. Each has an associated weight of one. The rat and mouse toxicity thresholds are based on the distribution of in-scope values in the focused WDI, as is the mutagenicity threshold. The hepatotoxicity rule "HEPX" reflects the way actual blood test results are interpreted, e.g., hepatotoxicity is indicated if aspartic acid transaminase (Ser_AST) and alanine transaminase (Ser_ALT) are both elevated in serum. Liver injury also usually elevates serum levels of lactate dehydrogenase and including Ser_LDH in the rule reduces the number of false positives. Concomitant elevation of the other serum enzyme model predictions, Ser_AlkPhos or Ser_GGT, is indicative of even more severe liver injury.

Table S5. *The five rules based on descriptors and predicted properties used to asses toxicity risk.*

Parameter	Rule of toxicity risk
**hERG**	hERG_FILTER = Yes and hERG_pIC$_{50}$ > [5.5,6.0] (potential hERG liability)
**rat**	*Rat_ Acute* < [200,300] (acute toxicity in rats)
**Xm:**	*Mouse_TD50* < [25,40] (carcinogenicity in chronic mouse studies)
**HEPX**	*Ser_AST* = Elevated and *Ser_ALT* = Elevated and<br>*Ser_LDH* = Elevated (liver enzymes elevated in serum)
**MUT**	*MUT_Risk* > 1

hERG, human Ether-a-go-go Related Gene***; hERG_FILTER,*** qualitative estimation of the likelihood of the hERG potassium channel inhibition in human ***hERG_pIC$_{50}$,*** a measure of affinity towards hERG K+channel and potential for cardiac toxicity [mol/L], ***Rat_acute***, LD$_{50}$ for lethal rat acute toxicity by any mechanism [mg/kg] ***Xm***, carcinogenicity in chronic mouse studies, ***Mouse_TD$_{50}$,*** the TD$_{50}$ is the dose of a substance given to mice orally throughout their lifetimes resulting in half of the population experiencing tumors [mg/kg/day]; ***HEPX***, Hepatotoxicity; ***Ser_AST*** serum aspartic acid transaminase, ***Ser_ALT*** serum alanine transaminase; ***Ser_LDH*** serum lactate dehydrogenase

2.1.4. Mutation risk

The mutation risk component of toxicity risk and ADMET risk integrates the 10 test predictions from Simulation Plus *in silico* Ames tests for mutagenicity. It exceeds 1.0 for 15% of the focused WDI subset and exceeds 1.2 for 9% of it. Most of the individual toxicity risk rules are rather conservative, with each individual rule being violated to some extent by 7-16% of in-scope predictions for WDI reference set. Toxicity risk is greater than 2.0 for 6% of the focused WDI after default out-of-scope risk penalties have been factored in; it is equal to 2.0 for 10% of it.

2.1.5. Summary of ADMET parameters and recommended ranges

The criteria used to set the ADMET scores (**Table S5**) are based on predictions for a refined reference subset drawn from the WDI and developed in ADMET modules by Simulation Plus (all the cited criteria and their scores are found in the manual provided by ADMET Predictor®). **Table S6** shows the recommended ranges of ADMET parameters.

Table S6. *Overview of ADMET parameters with their recommended ranges.*

Parameter	Recommended Range	Comments
**ADMET_Risk**	< 7	Includes components of all risk models as well as fraction unbound to plasma and volume of distribution.
**Absn_Risk**	< 4	Considers size, rotational bonds, hydrogen bonding capacity, polar surface area, permeability, lipophilicity, solubility.

**TOX_Risk**	**< 2**	Consists of hERG, acute toxicity in rats, carcinogenicity in chronic rat/mouse studies, hepatotoxicity, mutation.
**MUT_Risk**	**< 2**	Comprises of Ames mutagenicity test, which tests for mutagenic capacity of compounds in bacteria.
**CYP_Risk**	**< 2**	Includes inhibition of CYPs 1A2, 2C19, 2C9, 2D6, and 3A4, excessive clearance, and inhibition of midazolam or testosterone.

2.2. Transporter Models

The Transporter Module provides models for the estimation of the likelihood that a compound is a substrate of nine transporters that are responsible for most DDIs: the organic anion transport polypeptides 1B1 (**OATP1B1**; SLCo1B1) and 1B3 (**OATP1B3**; SLCo1B3), the organic anion transporters 1 (**OAT1**; SLC22A6) and 3 (**OAT3**; SLC22A8), the organic cation transporter 1 (**OCT1**; SLC22A1), **OCT2** (SLC22A2), the efflux transporter **P-gp** (which stands for permeability glycoprotein), the Breast Cancer Resistance Protein (**BCRP**), and the bile salt export pump (**BSEP**; ABCB11). The substrate categorization data were collected from various academic and commercial databases. ADMET Predictor® also offers nine different classification models for predicting how likely a compound is to inhibit a transporter. They cover two efflux transporters (P-gp and BCRP) and seven uptake transporters: OATP1B1, OATP1B3, OAT1, OAT3, OCT1, OCT2, and BSEP. A regression model - BSEP_IC50- provides quantitative estimates of inhibitory potency for BSEP.

2.3. Prediction of metabolites

ADMET Predictor uses ANNE-based substrate classification and site of metabolism (SOM) models to pick out atoms subject to CYP and AOX oxidation and then applies a set of transformations to those atoms to generate the initial oxidation product as well as subsequent spontaneous and enzymatic transformations.
