# Supplementary figures and images for: 3-CMC: Acute Effects in Male and Female Mice, Human Intoxication Case Series (Italy, 2014–2025), and Prediction of ADMET Properties
Source: Int J Mol Sci. 2025 Nov 29;26(23):11600. doi: 10.3390/ijms262311600 (PMC12692235; doi:10.3390/ijms262311600)

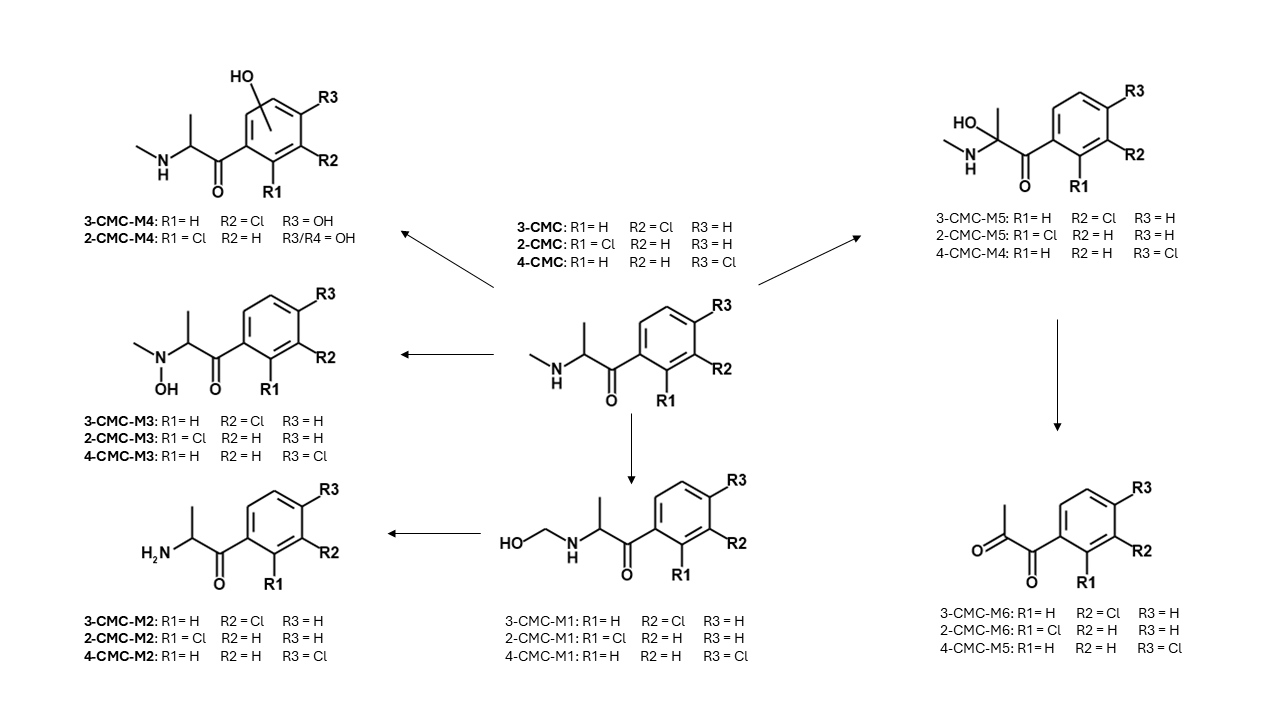

Supplement: Supplementary file 1 [file ijms-26-11600-s001.zip › Figure S1.tif]

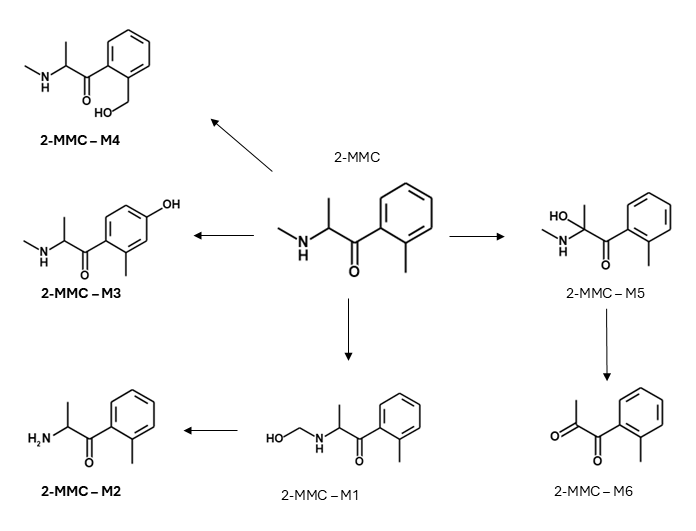

Supplement: Supplementary file 1 [file ijms-26-11600-s001.zip › Figure S2.tif]
